# Identification of Risk Factors and Symptoms of COVID-19: Analysis of Biomedical Literature and Social Media Data

**DOI:** 10.2196/20509

**Published:** 2020-10-02

**Authors:** Jouhyun Jeon, Gaurav Baruah, Sarah Sarabadani, Adam Palanica

**Affiliations:** 1 Klick Labs Klick Applied Sciences Toronto, ON Canada

**Keywords:** SARS-CoV-2, COVID-19, risk factor, symptom, diagnosis, treatment, biomedical literature, social media, Twitter, tweets

## Abstract

**Background:**

In December 2019, the COVID-19 outbreak started in China and rapidly spread around the world. Lack of a vaccine or optimized intervention raised the importance of characterizing risk factors and symptoms for the early identification and successful treatment of patients with COVID-19.

**Objective:**

This study aims to investigate and analyze biomedical literature and public social media data to understand the association of risk factors and symptoms with the various outcomes observed in patients with COVID-19.

**Methods:**

Through semantic analysis, we collected 45 retrospective cohort studies, which evaluated 303 clinical and demographic variables across 13 different outcomes of patients with COVID-19, and 84,140 Twitter posts from 1036 COVID-19–positive users. Machine learning tools to extract biomedical information were introduced to identify mentions of uncommon or novel symptoms in tweets. We then examined and compared two data sets to expand our landscape of risk factors and symptoms related to COVID-19.

**Results:**

From the biomedical literature, approximately 90% of clinical and demographic variables showed inconsistent associations with COVID-19 outcomes. Consensus analysis identified 72 risk factors that were specifically associated with individual outcomes. From the social media data, 51 symptoms were characterized and analyzed. By comparing social media data with biomedical literature, we identified 25 novel symptoms that were specifically mentioned in tweets but have been not previously well characterized. Furthermore, there were certain combinations of symptoms that were frequently mentioned together in social media.

**Conclusions:**

Identified outcome-specific risk factors, symptoms, and combinations of symptoms may serve as surrogate indicators to identify patients with COVID-19 and predict their clinical outcomes in order to provide appropriate treatments.

## Introduction

COVID-19 is an emerging infectious disease that has quickly spread worldwide. Since its outbreak in China in December 2019, over 4 million cases have been confirmed across more than 200 countries [1] (as of May 20, 2020), with the number of cases continuing to increase. Several studies have characterized possible symptoms (physical or mental features indicating a disease condition) and risk factors (variables associated with an increased risk of disease) for patients infected with COVID-19 [2,3]. However, the majority of retrospective studies have been based on a single outcome from a single center and counted the number of aggregate cases [4], providing a scattered and incomplete picture of the risk factors for disease severity. Furthermore, uncharacterized or uncommon symptoms have made COVID-19 difficult to diagnose, making it difficult to provide appropriate treatment to patients. Lack of a vaccine or optimized treatment raises the importance of early and definitive diagnosis for this disease. Additionally, there are limited hospital resources to triage patients based on symptoms to determine who is more or less likely to require intensive treatment (eg, intensive care unit [ICU] admission or intubation).

All of these uncertainties suggest there are urgent needs for a low-cost and efficient method of gathering COVID-19 symptom- and risk factor–related data as quickly as possible to reduce the medical and economic burden in our society. Instead of conducting time-consuming and costly clinical trials to examine patients, an alternative research avenue involves scraping public social media data to investigate potential risk factors of COVID-19 development. Social media provides an efficient method of gathering large amounts of representative data on the general public, in a cost-effective, scalable, and convenient manner any time of day, especially in remote or unattended regions. Here, we systematically investigated published biomedical literature to identify risk factors associated with outcomes of patients with COVID-19. We then gathered public Twitter data from COVID-19–positive users to expand the scientific literature and also examine rare or uncommon symptoms that were not previously well characterized.

## Methods

### Compiling Biomedical Literature on COVID-19, and Identifying Clinical and Demographic Variables

CORD-19 (COVID-19 Open Research Dataset) [5] was used to find biomedical literature on COVID-19. We compiled retrospective studies that investigated clinical and demographic variables in various outcomes of COVID-19. To do this, we collected literature published between January 2020 and March 2020. We then generated two sets of keywords. The first set of keywords represented cohort-based and retrospective studies, and comprised the following keywords: “epidemiological characteristics,” “clinical characteristics,” “risk factors,” “clinical features,” “cohort,” “clinical course,” “clinical findings,” “risk of death,” “pathological characteristics,” “retrospective,” and “mortality risk.” The second set represented COVID-19 (“novel coronavirus,” “coronavirus,” “COVID-19,” “SARS-COV-2,” “severe acute respiratory syndrome coronavirus 2,” “2019 novel coronavirus,” “2019-ncov,” and “coronavirus disease 2019”).

From the semantic analysis, we found 116 articles that contained both keywords and were likely to be relevant to our study. We next extracted 535 tables in those articles using Camelot [6] based on the notion that tables listed clinical and demographical variables, and their associated statistics. Articles without tabulated information (n=12) were removed. After careful manual curation of tables, reported data, and article themselves, 45 studies were chosen (Table S1 in Multimedia Appendix 1). In total, 304 clinical and demographic variables were evaluated in 45 studies. They were composed of 92 comorbidities/complications, 49 treatments, 124 lab findings, 34 symptoms, and 4 demographic variables (age, sex, alcohol drinking history, and smoking history). Literature collection was conducted in accordance with the PRISMA (Preferred Reporting Items for Systematic Reviews and Meta-Analysis) guidelines (Figure S1 in Multimedia Appendix 1) [7].

### Association Between Clinical and Demographic Variables and Clinical Outcomes

To explore the consistency between individual variables and clinical outcomes, we defined three types of associations. Positive associations indicated that a variable in the outcome group had a hazard or odd ratio >1, or a higher value with statistical significance (*P* value <.05) compared to the control group. In case a statistical test was not performed, we decided that there was a positive association when the outcome group had a 1.5-fold higher value than the control group. Negative association indicated that a variable in the outcome group had a hazard or odd ratio <1, or a lower value with statistical significance (*P* value <.05) compared to the control group. In case a statistical test was not performed, we decided that there was a negative association when the outcome group had a 1.5-fold lower value than the control group. When there was no significant change between the outcome group and the control group, the variable and clinical outcomes were assigned a “no association” designation. In terms of sex, when there were more males compared to females, we assumed there was a positive association with an outcome based on the case studies of sex and age of patients with COVID-19 in Italy [8] and New York City [9] (as of April 14, 2020). To identify outcome-specific risk factors in biomedical literature, we performed consensus analysis. Risk factors were the variables associated with an increased risk of disease or infection. Seven outcomes, which were studied at least twice, and 107 variables tested two or more times in studies were used for further analyses. Clinical and demographic variables with positive associations in >50% of studies, which investigated the same output, were defined as outcome-specific risk factors.

### Compiling Social Media Data and Identifying Symptoms of COVID-19–Positive Users

Twitter was used as the social media source. To identify COVID-19–positive users, we first collected users who used one of these phrases: “my positive COVID test,” “my positive COVID diagnosis,” “I am positive for COVID,” “I tested positive for COVID,” and “I have COVID-19” in their original tweets between January 2020 and March 2020. In total, 1036 users were identified as self-identified patients with COVID-19. We then collected additional tweets generated 14 days before and 14 days after the original tweets mentioning users’ COVID-19 status (n=84,140 tweets). To identify symptoms that were mentioned in tweets, we applied two symptom extraction methods. The Amazon Comprehend Medical tool [10] was applied to an entire set of tweets. Symptoms were physical or mental features indicating a disease condition. We considered two medical entities (symptoms and signs) as symptoms that users mentioned. We also implemented a symptom extraction model using Scispacy, version 0.2.4 (Python Software Foundation). Scispacy can handle scientific document and extracts medical and clinical terminology [11]. The model was trained on publicly available, domain-specific corpus of medical notes, which consists of 1500 PubMed articles with over 10,000 disease and related chemical terms. The model identifies medical name entities in tweet texts. We considered the medical entity “disease” as a symptom that users mentioned. In total, 51 symptoms from 574 COVID-19–positive users were identified from both symptom identification methods.

## Results

### Landscape of Clinical and Demographic Variables of COVID-19 in Biomedical Literature

**Figure 1:**
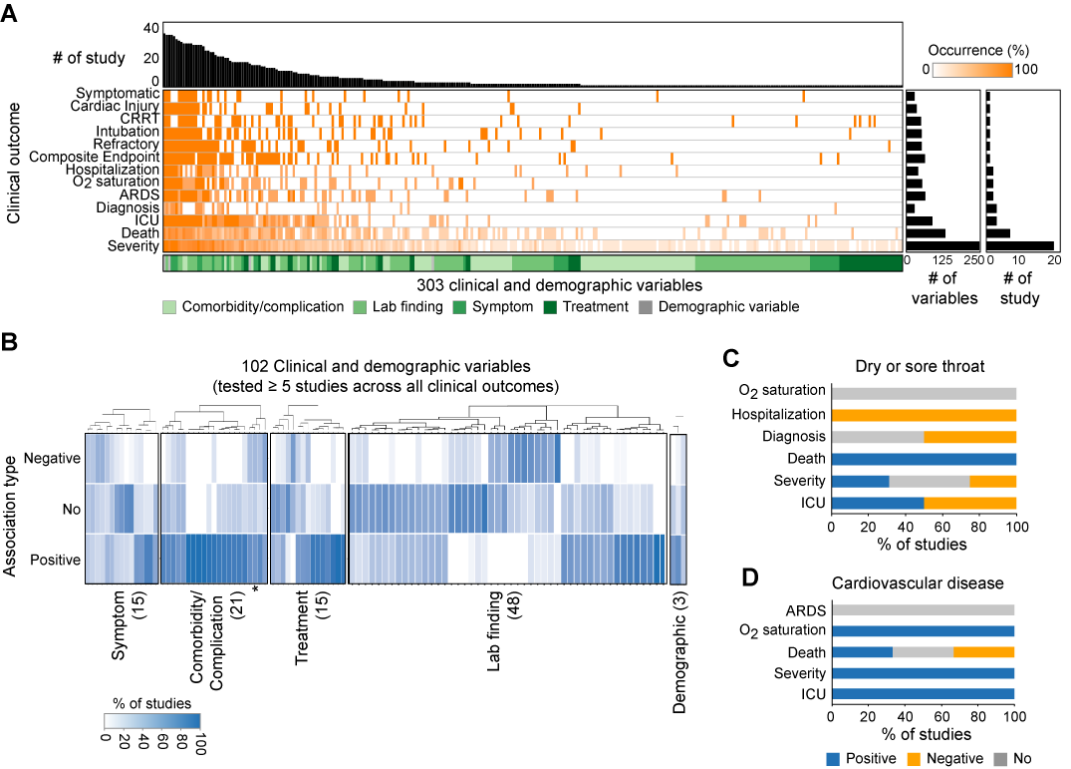
Properties of clinical and demographic variables of COVID-19. (A) Landscape of clinical and demographic variables. (B) Association between variables and overall clinical outcomes. Number of variables tested in ≥5 studies appears in brackets. Asterisk indicated association types of cancer. (C) and (D) Association types of clinical outcomes of COVID-19; association types of (C) dry or sore throat and (D) cardiovascular disease depending on different clinical outcomes were shown. CRRT: continuous renal replacement therapy; ARDS: acute respiratory distress syndrome; ICU: intensive care unit.

To understand the clinical and demographic properties of COVID-19, we performed a meta-analysis of 45 recently published biomedical studies (Figure 1A, and Table S1 in Multimedia Appendix 1). The literature evaluated 299 clinical variables (92 comorbidities/complications, 124 laboratory findings, 49 treatment options, and 34 symptoms) and 4 demographic variables (age, sex, alcohol drinking history, and smoking history) in 13 clinical outcomes. Seven outcomes (disease severity, death, ICU admission, diagnosis, acute respiratory distress syndrome [ARDS], O_2_ saturation, and hospitalization) were studied at least twice (Figure 1). On average, each study examined 72 variables, and 102 variables were assessed in at least five studies. Age and sex were measured in more than 80% of the studies. Diabetes and hypertension were the most commonly measured comorbidities (>50% of studies). Fever, cough, myalgia/fatigue, chest tightness/dyspnea, diarrhea, and headache/dizziness were the most commonly measured symptoms (>50% of studies). Eleven laboratory test values that measured liver and kidney function (eg, alanine aminotransferase and aspartate aminotransferase) and hematologic index (eg, lymphocytes, platelets, and neutrophils) were examined in >50% of studies. Therapy involving antiviral agents, antibiotics, and oxygen inhalation were used in more than 30% of the studies (Table S2 in Multimedia Appendix 1).

We next investigated the association between identified variables and clinical outcomes of patients with COVID-19. We considered 102 frequently tested variables (≥5 studies), and examined the proportion of studies that showed positive, negative, and no associations between clinical outcomes and a given variable (Table S3 in Multimedia Appendix 1). Positive associations indicated higher values (eg, disease severity increases as patients get older), while negative associations indicated lower values (eg, disease severity increases as basophil count decreases) of variables associated with clinical outcomes (see Methods for details). A no-association designation indicated there was no relation between variables and outcomes.

We found that the majority of variables (n=95, 93%) had inconsistent associations across clinical outcomes showing mixed association types (Figure 1B). Of those, 46 (45%) variables had all three types of associations. For example, cancer showed positive, negative, and no associations in 58% (n=11), 26% (n=3), and 16% (n=5) of studies, respectively (marked with an asterisk in Figure 1B). In total, 43% (9/21) of comorbidity/complication, 40% (6/15) of treatment, and 73% (11/15) of symptom variables showed all three types of associations. Laboratory findings showed relatively more consistent associations with clinical outcomes: 38% (18/48) of variables showed all association types. Furthermore, we found that variables had unique association types depending on different clinical outcomes. Dry or sore throat, one known symptom of COVID-19, showed positive, negative, and no associations in death, hospitalization, and O_2_ saturation, respectively (Figure 1C). Meanwhile, it showed mixed associations with other clinical outcomes, such as disease severity, ICU admission, and diagnosis. Cardiovascular disease, one common comorbidity of COVID-19, showed mixed associations in death, positive association with ICU and disease severity, but no association with ARDS (Figure 1D).

### Consensus Identification of Outcome-Specific Clinical and Demographic Variables

**Figure 2:**
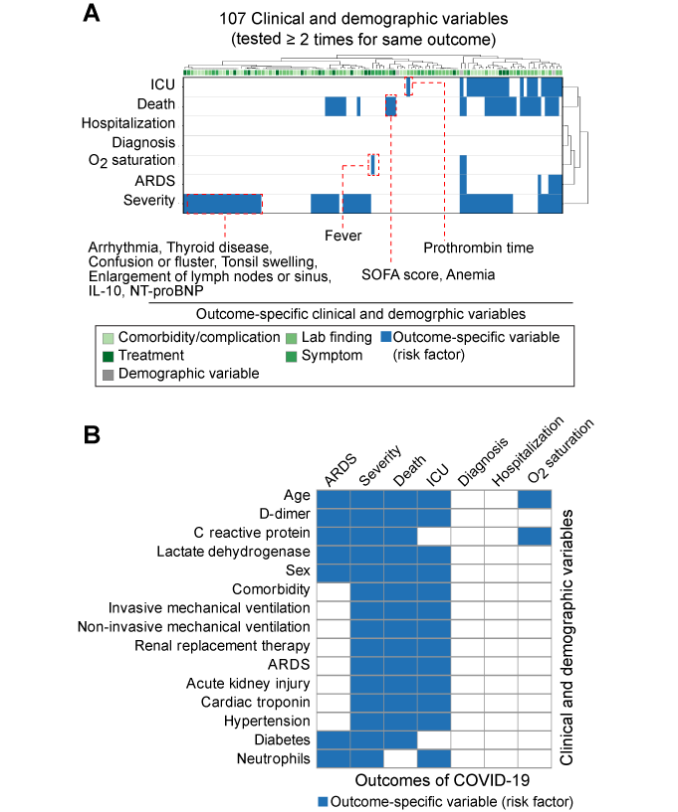
Consensus identification of outcome-specific clinical and demographic variables. (A) Outcome-specific clinical and demographic variables in a given outcome of COVID-19. Variables that were only specific for one clinical outcome were shown in the red-dashed box. Clinical and demographic variables that were specific for at least three outcomes are presented in (B). Blue coloring indicates identified outcome-specific variables (risk factors). ICU: SOFA: Sequential Organ Failure Assessment; ARDS: acute respiratory distress syndrome; IL-10: interleukin 10; NT-proBNP: N-terminal pro-brain natriuretic peptide.

According to biomedical literature at the time of publication, there were no effective treatment options, or well-identified symptoms, comorbidities, and lab findings to predict outcomes of COVID-19. Therefore, it seemed relevant to find a set of clinical and demographic variables that were specific for individual outcomes for better guidance on disease detection, treatment, and control. To generalize the importance of clinical and demographic variables, a consensus (level of agreement) analysis was performed. We collected 107 variables that were tested at least twice in a given outcome and defined them as outcome-specific risk factors when they showed positive associations with a given outcome in more than half of studies (Figure 2, and Table S4 in Multimedia Appendix 1). In total, we characterized 72 outcome-specific risk factors from the literature. As shown in Figure 2A, different sets of variables were specifically associated with individual outcomes. Arrhythmia, thyroid disease (comorbidity/complication), confusion/fluster, tonsil swelling, enlargement of lymph nodes/sinus (symptom), and levels of interleukin (IL)-10 and N-terminal pro-brain natriuretic peptide (NT-proBNP; lab finding) were specifically associated with the severity of disease progression. Level of prothrombin time was a specific risk factor for ICU admission. For death, Sequential Organ Failure Assessment (SOFA) score (lab finding) and anemia (symptom) showed positive associations. Fever was specifically associated with O_2_ saturation. We also observed 15 variables that were associated with several clinical outcomes (≥3 outcomes; Figure 2B). Age was a risk factor for ARDS, disease severity, death, ICU admission, and O_2_ saturation, but not for diagnosis and hospitalization. Sex (male) was a specific variable for ARDS, disease severity, death, and ICU admission. Three lab findings (D-dimer, C reactive protein, and lactate dehydrogenase) were specifically associated with four outcomes. Diabetes and hypertension (comorbidity/complication) were specific risk factors for disease severity and death. Identified outcome-specific variables could be surrogate risk factors to identify patients with COVID-19 and determine their treatment options.

### Expanding the COVID-19 Symptom Landscape and Identifying Novel Symptoms Using Social Media

**Figure 3:**
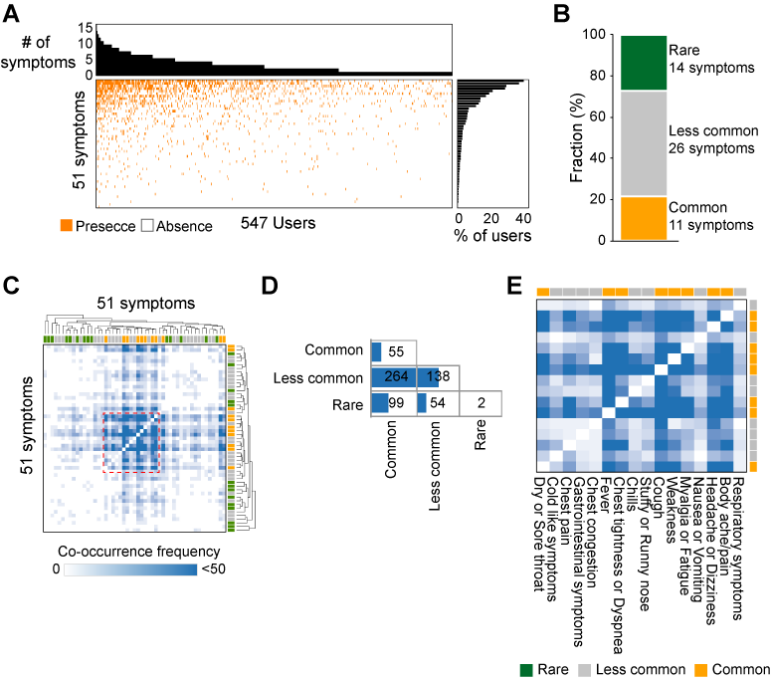
COVID-19–related symptoms extracted from social media data. (A) Landscape of symptoms identified from social media data. Orange and white indicate the presence and absence of symptoms in a given user, respectively. (B) Fraction of common, less common, and rare symptoms. Common symptoms were mentioned from >10% of users, and rare symptoms were mentioned from <1% of users. (C) Co-occurrence of symptoms. One major cluster was shown in the red-dashed box. (D) Number of symptoms pairs depending on mentioning frequency. Blue bars (bottom) indicate the number of co-occurring pairs. (E) One major cluster of symptom pairs. Green, gray, and orange indicate rare, less common, and common symptoms, respectively.

Early identification of symptoms is important for the successful treatment of disease [12]. Although COVID-19 showed heterogeneous and uncharacterized symptoms, a limited number of symptoms known to be associated with infectious diseases, such as fever, cough, and fatigue, were considered in the biomedical literature. Social media can provide rapid and efficient surveillance of disease risk and outbreaks [13,14]. Therefore, we decided to expand the symptom landscape by integrating social media data with biomedical literature. We first identified 1036 twitter users who introduced themselves as COVID-19–positive patients, and selected 574 users (55%) who openly and voluntarily discussed their COVID-19 symptoms (see Methods for details). In total, 51 symptoms were identified in social media data (Figure 3A, and Table S5 in Multimedia Appendix 1). On average, individual users mentioned 3 different symptoms (range 1-15). We grouped 51 symptoms into three categories based on their frequency of mention (Figure 3B). Common symptoms (n=11) were mentioned by >10% of users. Many were nonspecific symptoms of respiratory infections, such as fever, cough, and chest tightness/dyspnea (Table S5 in Multimedia Appendix 1). However, 14 symptoms were potentially COVID-19–specific and rarely reported in social media (ie, in <1% of users). Sputum, dehydration, anemia, urination problem, hair loss, enlargement of lymph nodes or sinus, and oral problems (eg, abrasions in mouth, mouth ulcers, sensitive teeth, toothache, and dry mouth) were defined as rare symptoms. In all, 26 symptoms were observed in 1% to 10% of users (less common). They included chills, chest pain, gastrointestinal symptoms, arthritis, anorexia, allergy-like symptoms, dyssomnias, ear- and eye-related problems, and skin problems such as blister, dry skin, chapped lips, rash, and itching. We next examined the symptoms that were mentioned together in social media (Figure 3C). We identified 612 co-occurring symptom pairs (Table S6 in Multimedia Appendix 1). In total, 264 (43%) symptoms ranged from common to less common (Figure 3D). One major cluster of symptoms was frequently mentioned together (Figure 3E). They were composed of 8 common symptoms, such as dry or sore throat, fever, chest tightness/dyspnea, cough, weakness, myalgia/fatigue, headache/dizziness, and body ache/pain (eg, neck and back pain and general body ache), and 8 less common symptoms, such as cold-like symptoms, chest pain and congestion, gastrointestinal symptoms, chills, stuffy or runny nose, nausea/vomiting, and respiratory symptoms such as lung pain. In total, 99 (16%) pairs were between common symptoms and rare symptoms. Cough, fever, headache/dizziness and body ache/pain (common symptoms) co-occurred with sputum, dehydration, and anemia at least 10 times (Table S6 in Multimedia Appendix 1). There were also two pairs of rare symptoms (Figure 3D): anemia–weight gain and anemia–urination problems (eg, urinary retention and weakened bladder).

**Figure 4:**
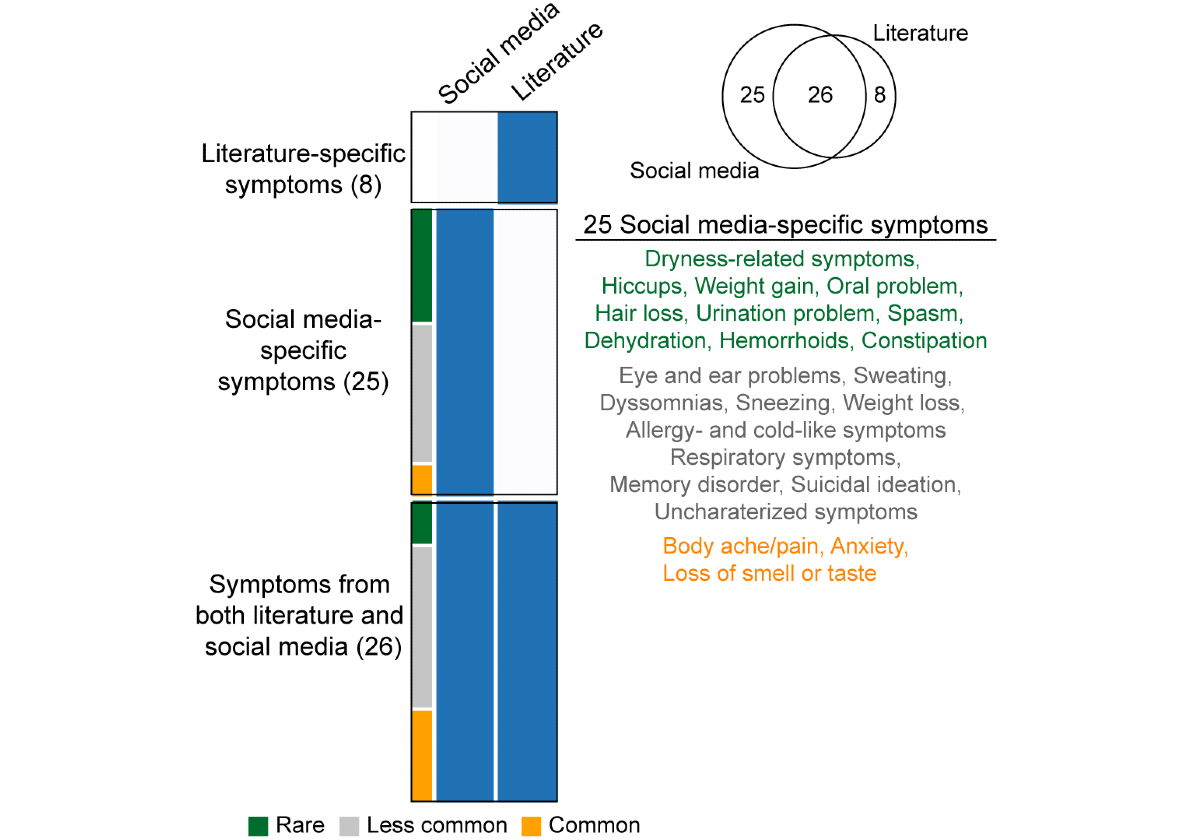
Identification of novel symptoms of COVID-19, and comparison of symptoms between biomedical literature and social media data. Symptoms that were observed in the literature or social media are colored in blue; 25 social media–specific symptoms are presented. Green, gray, and orange indicated rare, less common, and common symptoms, respectively.

Finally, we identified novel symptoms potentially related to COVID-19. We first extended the repertoire of symptoms by integrating social media data with biomedical literature. In total, 59 symptoms were identified (Figure 4, and Table S7 in Multimedia Appendix 1). Indeed, social media identified more symptoms (n=51) than the literature (n=34), and most symptoms (26/34, 76%) in the literature were equally observed in social media. Interestingly, 25 (42%) were novel symptoms that were only mentioned in social media and were not considered in the literature. Loss of smell or taste, and body ache/pain were frequently mentioned common symptoms in social media. Problems involving eyes (eg, dry eye and eye pain) and ears (eg, ear pain and earache), sweating, sneezing, and allergy-like symptoms were mentioned at a moderate frequency only in social media. Of 14 rare symptoms, 10 were only observed in social media: dehydration, dryness-related symptoms (eg, feeling dry), oral problem, hair loss, urination problem, spasm, hemorrhoids, constipation, hiccups, and weight gain. These social media–specific rare symptoms would be potential novel symptoms for COVID-19, when we considered that 4 rare symptoms (ie, abdominal pain, anemia, sputum, and enlargement of lymph nodes or sinus) were already evaluated in the literature (Figure 4).

## Discussion

### Principal Findings

Our meta-analysis based on biomedical literature showed the inconsistency of clinical and demographic variables across clinical outcomes. From the consensus analysis, we identified outcome-specific risk factors that may be helpful to identify patients and predict their specific clinical outcomes. Social media data expanded the repertoire of COVID-19 symptoms from biomedical literature. In addition to more commonly reported symptoms, social media data revealed loss of smell or taste, body ache/pain, and back/neck pain, as well as other less common and rare symptoms such as urination problems, dehydration, allergy-like symptom, and ear- and eye-related problems. Indeed, loss of smell or taste was recently proposed as one of the key features of a COVID-19 diagnosis model [15], and body ache/pain was suggested by the Centers for Disease Control and Prevention. COVID-19 is an ongoing health problem, and the symptoms that medical institutes and ordinary people should consider will be evolving as more studies are published and social media data are explored. This evolution may help improve the definitions of symptom types (common, less common, and rare symptoms) and social media–specific symptoms.

From social media data, we observed that certain combinations of symptoms were frequently observed among patients with COVID-19. Interestingly, we identified two pairs composed of rare symptoms (anemia–weight gain and anemia–urination problems, such as urination retention and weakened bladder). It has been shown that COVID-19 attacks hemoglobin in red blood cells and restricts oxygen transportation [16]. A persistent reduction of oxygen transportation leads to the development of anemia [17]. Urinary bladder is enriched with ACE2 positive cells and proposed as a target organ for COVID-19 invasion [18]. Our results highlighted that symptom combinations could guide the reliable identification of patients with COVID-19 rather than a single common symptom that may result in false positives.

### Limitations and Future Work

One of the limitations of our study is the self-reported nature of social media data and the lack of more detailed information from the patients. We observed that 55% of social media users who were self-identified patients with COVID-19 mentioned symptoms, and an additional 8% mentioned potential comorbidities. Thus, only 63% of social media users communicated information on COVID-19 conditions, which means that at least 37% of users could be either false positives (they were not COVID-19–positive users) or asymptomatic patients. Alternatively, it is possible that we have not captured all COVID-19–positive patients in our social media collection due to the limited amount of keyword searches. Nevertheless, various studies have indicated that between 4% and 78% of all COVID-19–positive patients were asymptomatic [19], and this seems to vary widely based on age of patients, test location, and time of testing after infection [20-23]. Thus, our research is in line with other studies demonstrating the vast range of patients with COVID-19 who show or report no symptoms. It should also be noted that Twitter was the source of the social media data we examined, and perhaps more symptoms would be discovered if we analyzed other sources. Twitter does have a wide, representative user base around the world, and provides open source information that can be easily gathered, but future research could examine alternate social media sources.

Although social media may lack depth of patient information, it provides an effective method of collecting breadth of data. Social media data can be gathered noninvasively across the world, 24 hours a day, and is an extremely efficient method [24] for rapidly disseminating new knowledge related to COVID-19. In other words, clinicians and scientists can collect new patient information from various regional locations, as well as quickly circulate public service announcements for a wide range of audiences. Social media hubs provide a useful alternate source of patient data to explore clinical characteristics of various disease states and populations.

Another limitation of our research involves the limited number of available biomedical studies on individual outcomes. Of the 13 reported clinical outcomes for COVID-19, 7 were studied at least two times, limiting opportunities to perform more systematic and consensus analyses of the landscape of risk factors and symptoms. In fact, we observed there was no significant risk factors associated with diagnosis and hospitalization indicating the current lack of clinical understanding at the early stage of COVID-19. Use of additional literature that will be published in the future and electronic health record studies [25] may refine the assessment of risk factors and symptoms, and increase the accuracy of patient identification for different clinical outcomes.

### Conclusion

In this meta-data study, we demonstrated the extensive variability present in clinical and demographic variables across COVID-19 outcomes, and the usefulness of gathering social media data as an effective and alternative way to uncover less common or other types of symptoms that have not been previously reported. Our findings show the practicality and feasibility of employing social media data for investigating new disease states. These practices could be incorporated into routine procedures for early COVID-19 identification and determination of clinical outcomes, in order to provide appropriate interventions and treatments.

## Appendix

Multimedia Appendix 1 Supplementary materials.

## Abbreviations

ARDS: acute respiratory distress syndrome
CORD-19: COVID-19 Open Research Dataset
ICU: intensive care unit
IL: interleukin
NT-proBNP: N-terminal pro-brain natriuretic peptide
PRISMA: Preferred Reporting Items for Systematic Reviews and Meta-Analysis
SOFA: Sequential Organ Failure Assessment
