# Supplementary material for: Identification of Risk Factors and Symptoms of COVID-19: Analysis of Biomedical Literature and Social Media Data
Source: J Med Internet Res. 2020 Oct 2;22(10):e20509. doi: 10.2196/20509 (PMC7537723; doi:10.2196/20509)
Supplement: Multimedia Appendix 1 [file jmir_v22i10e20509_app1.docx]

**Supplementary Figure and Tables**

**Identification of Risk Factors and Symptoms of COVID-19: Analysis of Biomedical Literature and Social Media Data**

Jouhyun Jeon^1, *^, Gaurav Baruah^1^, Sarah Sarabadani^1^, Adam Palanica^1^

^1.^ Klick Labs, Klick Applied Sciences, 175 Bloor Street East, Suite 300, Toronto, Ontario, Canada, M4W 3R8.

**Figure S1. PRISMA Flowchart**

**Table S1. List of biomedical literature**

**Table S2. List of clinical and demographic variables in biomedical literature**

**Table S3. Association between clinical and demographic variables and outcomes of COVID-19**

**Table S4. List of clinical and demographic variables in biomedical literature**

**Table S5. List of symptoms mentioned in social media**

**Table S6. Co-occurrence of symptoms in social media**

**Table S7. Novel COVID-19 related symptoms**

**Figure S1. PRISMA Flowchart**

**
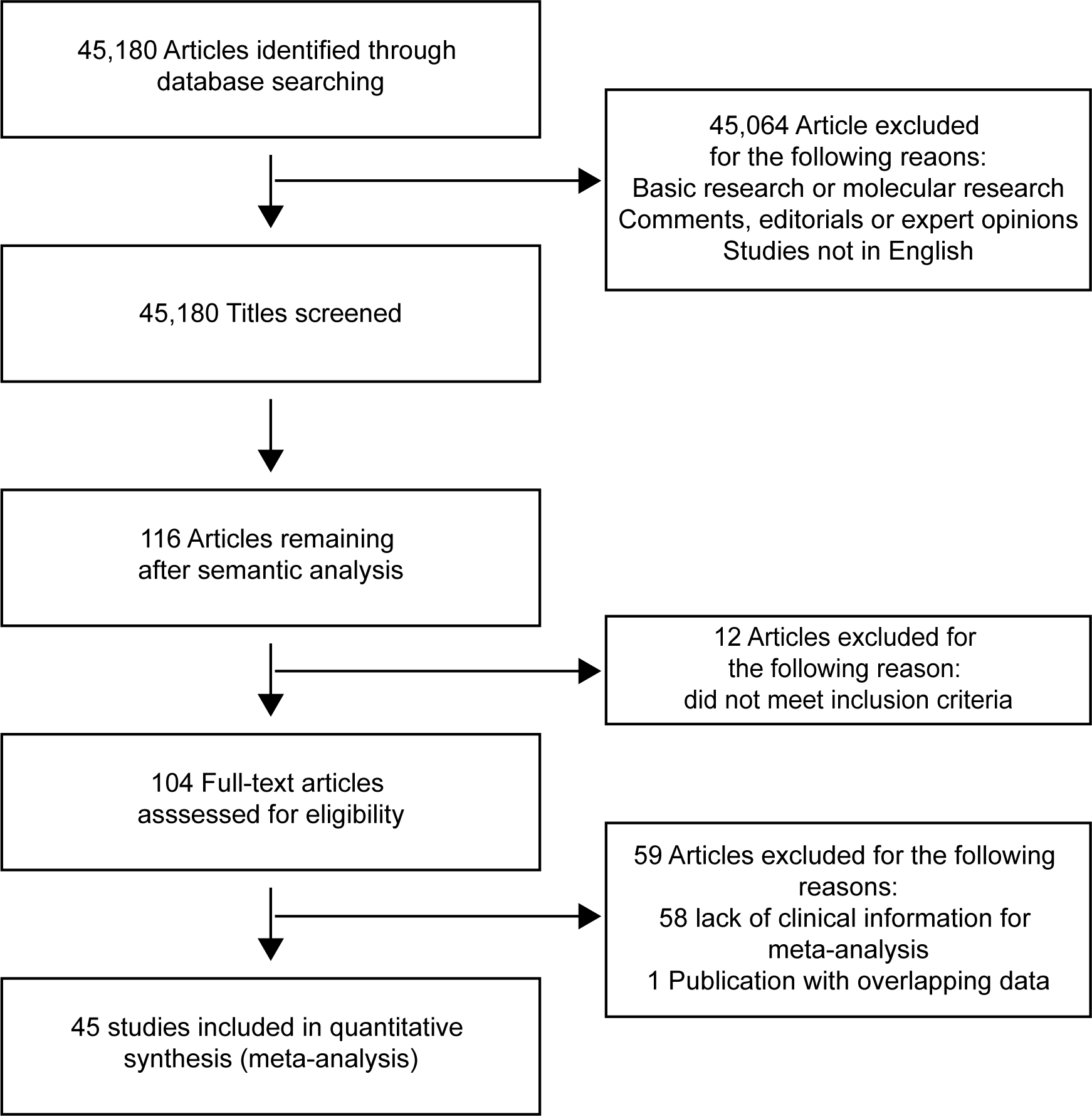
**

**Table S1. List of biomedical literature**

| LiteratureID | Outcome | Title | doi |
| --- | --- | --- | --- |
| 5l6c0it4 | ARDS | Risk Factors Associated With Acute Respiratory Distress Syndrome and Death in Patients With Coronavirus Disease 2019 Pneumonia in Wuhan, China | 10.1001/jamainternmed.2020.0994 |
| mlkxcn5k | ARDS | Clinical features and progression of acute respiratory distress syndrome in coronavirus disease 2019 | 10.1101/2020.02.17.20024166 |
| qbxx6yae | CardiacInjury | Clinical features and outcomes of 2019 novel coronavirus-infected patients with cardiac injury | 10.1101/2020.03.11.20030957 |
| umttnp1g | CompositeEndpoint | Clinical Characteristics of Coronavirus Disease 2019 in China | 10.1056/nejmoa2002032 |
| nnlynjoy | CRRT | Effect of continuous renal replacement therapy on all-cause mortality in COVID-19 patients undergoing invasive mechanical ventilation: a retrospective cohort study | 10.1101/2020.03.16.20036780 |
| 5l6c0it4 | Death | Risk Factors Associated With Acute Respiratory Distress Syndrome and Death in Patients With Coronavirus Disease 2019 Pneumonia in Wuhan, China | 10.1001/jamainternmed.2020.0994 |
| di7hfghi | Death | Clinical course and outcomes of critically ill patients with SARS-CoV-2 pneumonia in Wuhan, China: a single-centered, retrospective, observational study | 10.1016/s2213-2600(20)30079-5 |
| igfur3l9 | Death | Influence factors of death risk among COVID-19 patients in Wuhan, China: a hospital-based case-cohort study | 10.1101/2020.03.13.20035329 |
| jir7n19b | Death | Clinical features and outcomes of 221 patients with COVID-19 in Wuhan, China | 10.1101/2020.03.02.20030452 |
| k36rymkv | Death | Clinical course and risk factors for mortality of adult inpatients with COVID-19 in Wuhan, China: a retrospective cohort study | 10.1016/s0140-6736(20)30566-3 |
| vg1vvm6f | Death | Clinical characteristics of 101 non-surviving hospitalized patients with COVID-19: A single center, retrospective study | 10.1101/2020.03.04.20031039 |
| zblitbo0 | Death | Myocardial injury is associated with in-hospital mortality of confirmed or suspected COVID-19 in Wuhan, China: A single center retrospective cohort study | 10.1101/2020.03.21.20040121 |
| cr0s5d1j | Diagnosis | Prevalence and clinical features of 2019 novel coronavirus disease (COVID-19) in the Fever Clinic of a teaching hospital in Beijing: a single-center, retrospective study | 10.1101/2020.02.25.20027763 |
| p5ecpcll | Diagnosis | Analysis on the Clinical Characteristics of 36 Cases of Novel Coronavirus Pneumonia in Kunming | 10.1101/2020.02.28.20029173 |
| rdgyej4p | Diagnosis | Clinical Characteristics of 2019 Novel Infected Coronavirus PneumoniaÔºöA Systemic Review and Meta-analysis | 10.1101/2020.02.14.20021535 |
| 011k6mm0 | Hospitalization | Evaluation of the clinical characteristics of suspected or confirmed cases of COVID-19 during home care with isolation: A new retrospective analysis based on O2O | 10.1101/2020.02.26.20028084 |
| w4zad1zt | Hospitalization | Epidemiological and Clinical Characteristics of 17 Hospitalized Patients with 2019 Novel Coronavirus Infections Outside Wuhan, China | 10.1101/2020.02.11.20022053 |
| 0nhgxoim | ICU | Clinical features of patients infected with 2019 novel coronavirus in Wuhan, China | 10.1016/s0140-6736(20)30183-5 |
| 6yinwbfh | ICU | Clinical Characteristics of 138 Hospitalized Patients With 2019 Novel Coronavirus-Infected Pneumonia in Wuhan, China | 10.1001/jama.2020.1585 |
| dmud2zf7 | ICU | Clinical Features of Patients Infected with the 2019 Novel Coronavirus (COVID-19) in Shanghai, China | 10.1101/2020.03.04.20030395 |
| 9ecuca17 | Intubation | Clinical findings in critical ill patients infected with SARS-Cov-2 in Guangdong Province, China: a multi-center, retrospective, observational study | 10.1101/2020.03.03.20030668 |
| h9hjslvb | Refractory | Clinical characteristics of refractory COVID-19 pneumonia in Wuhan, China | 10.1093/cid/ciaa270 |
| 5ciaonf0 | Severity | Epidemiological and clinical features of 291 cases with coronavirus disease 2019 in areas adjacent to Hubei, China: a double-center observational study | 10.1101/2020.03.03.20030353 |
| 5xyt8d5u | Severity | Clinical characteristics of 140 patients infected by SARS-CoV-2 in Wuhan, China | 10.1111/all.14238 |
| ba1br2a0 | Severity | CT manifestations of coronavirus disease-2019: A retrospective analysis of 73 cases by disease severity | 10.1016/j.ejrad.2020.108941 |
| bsgqsdbx | Severity | Clinical and immunologic features in severe and moderate forms of Coronavirus Disease 2019 | 10.1101/2020.02.16.20023903 |
| dbzrd23n | Severity | A descriptive study of the impact of diseases control and prevention on the epidemics dynamics and clinical features of SARS-CoV-2 outbreak in Shanghai, lessons learned for metropolis epidemics prevention | 10.1101/2020.02.19.20025031 |
| ddq2q1pg | Severity | Epidemiological and clinical characteristics of COVID-19 in Shenzhen, the largest migrant city of China | 10.1101/2020.03.22.20035246 |
| g70y02rk | Severity | Epidemiological, clinical characteristics of cases of SARS-CoV-2 infection with abnormal imaging findings | 10.1016/j.ijid.2020.03.040 |
| glq0lckz | Severity | Clinical Characteristics of SARS-CoV-2 Pneumonia Compared to Controls in Chinese Han Population | 10.1101/2020.03.08.20031658 |
| j0ufth5d | Severity | Early Prediction of Disease Progression in 2019 Novel Coronavirus Pneumonia Patients Outside Wuhan with CT and Clinical Characteristics | 10.1101/2020.02.19.20025296 |
| jir7n19b | Severity | Clinical features and outcomes of 221 patients with COVID-19 in Wuhan, China | 10.1101/2020.03.02.20030452 |
| jtsxpjhc | Severity | Clinical features and laboratory inspection of novel coronavirus pneumonia (COVID-19) in Xiangyang, Hubei | 10.1101/2020.02.23.20026963 |
| jxtch47t | Severity | Epidemiologic and Clinical Characteristics of 91 Hospitalized Patients with COVID-19 in Zhejiang, China: A retrospective, multi-centre case series | 10.1101/2020.02.23.20026856 |
| mlkxcn5k | Severity | Clinical features and progression of acute respiratory distress syndrome in coronavirus disease 2019 | 10.1101/2020.02.17.20024166 |
| nsrm0axa | Severity | Epidemiological and clinical features of 2019-nCoV acute respiratory disease cases in Chongqing municipality, China: a retrospective, descriptive, multiple-center study | 10.1101/2020.03.01.20029397 |
| qdmuhxaw | Severity | Clinical Characteristics of Coronavirus Disease 2019 in Hainan, China | 10.1101/2020.03.19.20038539 |
| qlkt5fzp | Severity | Clinical Characteristics of Patients with Severe Pneumonia Caused by the 2019 Novel Coronavirus in Wuhan, China | 10.1101/2020.03.02.20029306 |
| std4jddn | Severity | COVID-19 Myocarditis and Severity FactorsÔºö An Adult Cohort Study | 10.1101/2020.03.19.20034124 |
| umttnp1g | Severity | Clinical Characteristics of Coronavirus Disease 2019 in China | 10.1056/nejmoa2002032 |
| yqiggzk9 | Severity | Clinical characteristics of 51 patients discharged from hospital with COVID-19 in ChongqingÔºåChina | 10.1101/2020.02.20.20025536 |
| yx8b2moc | Severity | Incidence, clinical characteristics and prognostic factor of patients with COVID-19: a systematic review and meta-analysis | 10.1101/2020.03.17.20037572 |
| 7k36owrf | O2 saturation | Clinical Features of 69 Cases with Coronavirus Disease 2019 in Wuhan, China \| Clinical Infectious Diseases \| Oxford Academic | 10.1093/cid/ciaa272 |
| m5k28kbu | O2 saturation | Epidemiologic Features and Clinical Course of Patients Infected With SARS-CoV-2 in Singapore | 10.1001/jama.2020.3204 |
| 289fu2yb | Symptomatic | Clinical features of imported cases of coronavirus disease 2019 in Tibetan patients in the Plateau area | 10.1101/2020.03.09.20033126 |

**Table S2. List of clinical and demographic variables in biomedical literature**

| Variable | num_study | Fraction (%) | Class |
| --- | --- | --- | --- |
| Abdominal_pain | 7 | 15.56 | Symptom |
| Acid_suppression_drugs | 1 | 2.22 | Treatment |
| Acidosis | 1 | 2.22 | Comorbidity/Complication |
| Activated_partial_thromboplastin_time | 8 | 17.78 | LaboratoryFinding |
| Acute_cardiac_injury | 8 | 17.78 | Comorbidity/Complication |
| Acute_kidney_injury | 11 | 24.44 | Comorbidity/Complication |
| Acute_liver_injury | 2 | 4.44 | Comorbidity/Complication |
| Acute_lung_injury | 1 | 2.22 | Comorbidity/Complication |
| Acute_respiratory_distress_syndrome | 13 | 28.89 | Comorbidity/Complication |
| Age | 37 | 82.22 | Demographic |
| Alanine_aminotransferase | 30 | 66.67 | LaboratoryFinding |
| Albumin | 21 | 46.67 | LaboratoryFinding |
| Albumin_Globulin_ratio | 1 | 2.22 | LaboratoryFinding |
| Alcoholic_hepatitis | 1 | 2.22 | Comorbidity/Complication |
| Allergic_rhinitis | 1 | 2.22 | Symptom |
| Anemia | 3 | 6.67 | Symptom |
| Anorexia | 15 | 33.33 | Symptom |
| Antibiotic_therapy | 15 | 33.33 | Treatment |
| Antifungal_therapy | 7 | 15.56 | Treatment |
| Antiviral_therapy | 17 | 37.78 | Treatment |
| Aortic_sclerosis | 1 | 2.22 | Comorbidity/Complication |
| APACHE_II | 4 | 8.89 | LaboratoryFinding |
| Appendectomy | 1 | 2.22 | Comorbidity/Complication |
| APTT | 5 | 11.11 | LaboratoryFinding |
| Arbidol | 5 | 11.11 | Treatment |
| Arrhythmia | 6 | 13.33 | Comorbidity/Complication |
| Arterial_pressure | 3 | 6.67 | LaboratoryFinding |
| Arthralgia | 2 | 4.44 | Symptom |
| Aspartate_aminotransferase | 29 | 64.44 | LaboratoryFinding |
| Aspartate_aminotransferase_Alanine_aminotransferase_ratio | 1 | 2.22 | LaboratoryFinding |
| Asthma | 2 | 4.44 | Comorbidity/Complication |
| Atopic_dermatitis | 1 | 2.22 | Symptom |
| Atrial_fibrillation | 1 | 2.22 | Comorbidity/Complication |
| Autoimmune_Diseases_or_Immunodeficiency | 2 | 4.44 | Comorbidity/Complication |
| B_Lymphocytes | 3 | 6.67 | LaboratoryFinding |
| B_type_natriuretic_peptide | 2 | 4.44 | LaboratoryFinding |
| Bacteraemia | 1 | 2.22 | Comorbidity/Complication |
| Bacterial_pneumonia | 1 | 2.22 | Comorbidity/Complication |
| Barotrauma | 1 | 2.22 | Comorbidity/Complication |
| Basophils | 2 | 4.44 | LaboratoryFinding |
| Blood_Coagulation_Disorders | 2 | 4.44 | Comorbidity/Complication |
| Blood_glucose | 2 | 4.44 | LaboratoryFinding |
| BMI | 2 | 4.44 | LaboratoryFinding |
| Brain_disease | 1 | 2.22 | Comorbidity/Complication |
| C_reactive_protein | 28 | 62.22 | LaboratoryFinding |
| Ca2__receptor_blocker | 1 | 2.22 | Treatment |
| Calcium | 4 | 8.89 | LaboratoryFinding |
| Cancer | 17 | 37.78 | Comorbidity/Complication |
| Cardiac_disease_and_injury | 6 | 13.33 | Comorbidity/Complication |
| Cardiac_intervention | 1 | 2.22 | Comorbidity/Complication |
| Cardiac_troponin | 17 | 37.78 | LaboratoryFinding |
| Cardio_pulmonary_resuscitation | 1 | 2.22 | Treatment |
| Cardiopathy | 1 | 2.22 | Comorbidity/Complication |
| Cardiovascular_disease | 20 | 44.44 | Comorbidity/Complication |
| CD28_CD4_T_cells_CD4_T | 1 | 2.22 | LaboratoryFinding |
| CD28_CD8_T | 1 | 2.22 | LaboratoryFinding |
| CD3 | 5 | 11.11 | LaboratoryFinding |
| CD4 | 7 | 15.56 | LaboratoryFinding |
| CD4_8 | 1 | 2.22 | LaboratoryFinding |
| CD45 | 1 | 2.22 | LaboratoryFinding |
| CD45RA__Treg | 1 | 2.22 | LaboratoryFinding |
| CD45RA_CD4_T_cells__CD4_T | 1 | 2.22 | LaboratoryFinding |
| CD45RO_CD4_T_cells__CD4_T | 1 | 2.22 | LaboratoryFinding |
| CD45RO_Treg | 1 | 2.22 | LaboratoryFinding |
| CD8 | 7 | 15.56 | LaboratoryFinding |
| Cerebrovascular_disease | 12 | 26.67 | Comorbidity/Complication |
| Cesarean_section | 1 | 2.22 | Comorbidity/Complication |
| Chest_congestion | 1 | 2.22 | Symptom |
| Chest_pain | 7 | 15.56 | Symptom |
| Chest_tightness_or_Dyspnea | 29 | 64.44 | Symptom |
| Chills | 6 | 13.33 | Symptom |
| Chinese_Medicine | 5 | 11.11 | Treatment |
| Chlamydia_pneumonia_antibody | 1 | 2.22 | LaboratoryFinding |
| Chloride | 4 | 8.89 | LaboratoryFinding |
| Chloroquine_or_Hydroxychloroquine | 2 | 4.44 | Treatment |
| Cholecystectomy | 1 | 2.22 | Comorbidity/Complication |
| Cholelithiasis | 1 | 2.22 | Comorbidity/Complication |
| Cholinesterase | 1 | 2.22 | LaboratoryFinding |
| Chronic_gastritis_and_gastric_ulcer | 1 | 2.22 | Comorbidity/Complication |
| Chronic_hepatitis | 3 | 6.67 | Comorbidity/Complication |
| Chronic_kidney_disease | 14 | 31.11 | Comorbidity/Complication |
| Chronic_liver_disease | 9 | 20.00 | Comorbidity/Complication |
| Chronic_obstructive_lung_disease | 15 | 33.33 | Comorbidity/Complication |
| Chronic_pharyngitis | 1 | 2.22 | Comorbidity/Complication |
| Chronic_pulmonary_disease | 2 | 4.44 | Comorbidity/Complication |
| Chronic_Respiratory_Disease | 1 | 2.22 | Comorbidity/Complication |
| CK_MB | 10 | 22.22 | LaboratoryFinding |
| Co_infection_of_Bacteria | 2 | 4.44 | Comorbidity/Complication |
| Co_infection_of_Fungus | 4 | 8.89 | Comorbidity/Complication |
| Co_infection_of_Other_viruses | 4 | 8.89 | Comorbidity/Complication |
| Coma | 1 | 2.22 | Symptom |
| Combined_treatment | 1 | 2.22 | Treatment |
| Comorbidity | 14 | 31.11 | Comorbidity/Complication |
| Complement_3 | 3 | 6.67 | LaboratoryFinding |
| Complement_4 | 3 | 6.67 | LaboratoryFinding |
| Confusion_or_Fluster | 2 | 4.44 | Symptom |
| Conjunctival_congestion | 1 | 2.22 | Symptom |
| Convalescent_plasma | 1 | 2.22 | Treatment |
| Corticosteroid | 7 | 15.56 | Treatment |
| Cough | 35 | 77.78 | Symptom |
| Craniocerebral_surgery | 1 | 2.22 | Comorbidity/Complication |
| Creatine_kinase | 24 | 53.33 | LaboratoryFinding |
| Creatinine | 30 | 66.67 | LaboratoryFinding |
| creatinine | 2 | 4.44 | LaboratoryFinding |
| Cumulative_fluid_balance_in_ICU | 1 | 2.22 | Treatment |
| CURB_65_score | 2 | 4.44 | LaboratoryFinding |
| Cystatin_C | 1 | 2.22 | LaboratoryFinding |
| D_dimer | 25 | 55.56 | LaboratoryFinding |
| Diabetes | 30 | 66.67 | Comorbidity/Complication |
| Diarrhea | 29 | 64.44 | Symptom |
| Diastolic_blood_pressure | 4 | 8.89 | LaboratoryFinding |
| Digestive_tracthemorrhage | 1 | 2.22 | Comorbidity/Complication |
| Direct_Bilirubin | 2 | 4.44 | LaboratoryFinding |
| Disseminated_intravascular_coagulation | 3 | 6.67 | Comorbidity/Complication |
| Diuretics | 1 | 2.22 | Treatment |
| Drinking | 3 | 6.67 | Demographic |
| Drug_hypersensitivity | 1 | 2.22 | Symptom |
| Dry_or_Sore_throat | 21 | 46.67 | Symptom |
| Dysbiosis | 1 | 2.22 | Comorbidity/Complication |
| ECMO | 8 | 17.78 | Treatment |
| Electrolyte_imbalance | 3 | 6.67 | Comorbidity/Complication |
| Enlargement_of_lymph_nodes_or_sinus | 2 | 4.44 | Symptom |
| Eosinophils | 6 | 13.33 | LaboratoryFinding |
| Epilepsy | 1 | 2.22 | Comorbidity/Complication |
| Eructation | 1 | 2.22 | Symptom |
| Erythrocyte_sedimentation_rate | 9 | 20.00 | LaboratoryFinding |
| Estimated_Glomerular_Filtration_Rate | 2 | 4.44 | LaboratoryFinding |
| Expectorant_or_Antitussive_drugs | 2 | 4.44 | Treatment |
| Fever | 36 | 80.00 | Symptom |
| Fibrinogen | 6 | 13.33 | LaboratoryFinding |
| Food_allergy | 1 | 2.22 | Symptom |
| Gastric_mucosa_repair_agent | 1 | 2.22 | Treatment |
| Gastrointestinal_disorder | 1 | 2.22 | Comorbidity/Complication |
| Gastrointestinal_haemorrhage | 2 | 4.44 | Comorbidity/Complication |
| Gastrointestinal_symptoms | 1 | 2.22 | Symptom |
| Globulin | 3 | 6.67 | LaboratoryFinding |
| Glucocorticoid_therapy | 11 | 24.44 | Treatment |
| Glucose | 3 | 6.67 | LaboratoryFinding |
| Glutathione_reductase | 1 | 2.22 | LaboratoryFinding |
| Glycosylated_hemoglobin | 1 | 2.22 | LaboratoryFinding |
| HCO3_ | 1 | 2.22 | LaboratoryFinding |
| Headache_or_Dizziness | 24 | 53.33 | Symptom |
| Heart_disease | 1 | 2.22 | Comorbidity/Complication |
| Heart_Failure | 3 | 6.67 | Comorbidity/Complication |
| Hemoglobin | 21 | 46.67 | LaboratoryFinding |
| Hemoptysis | 5 | 11.11 | Symptom |
| Hemorrhoidectomy | 1 | 2.22 | Comorbidity/Complication |
| Heparin | 1 | 2.22 | Treatment |
| Hepatic_abnormality | 1 | 2.22 | Comorbidity/Complication |
| Hepatitis_B_infection | 1 | 2.22 | Comorbidity/Complication |
| HIV_infection | 2 | 4.44 | Comorbidity/Complication |
| HLA_DR_CD8_T_cells__CD8_T | 1 | 2.22 | LaboratoryFinding |
| Hormone_Therapy | 1 | 2.22 | Treatment |
| HR | 11 | 24.44 | LaboratoryFinding |
| Hyperlipidemia | 2 | 4.44 | Comorbidity/Complication |
| Hypertension | 30 | 66.67 | Comorbidity/Complication |
| Hypoalbuminemia | 1 | 2.22 | Comorbidity/Complication |
| Hypoproteinemia | 2 | 4.44 | Comorbidity/Complication |
| Hysterectomy | 1 | 2.22 | Comorbidity/Complication |
| IFN_Œ≥ | 1 | 2.22 | LaboratoryFinding |
| IGA | 1 | 2.22 | LaboratoryFinding |
| IGG | 1 | 2.22 | LaboratoryFinding |
| IGM | 1 | 2.22 | LaboratoryFinding |
| IL_1 | 1 | 2.22 | LaboratoryFinding |
| IL_10 | 3 | 6.67 | LaboratoryFinding |
| IL_17A | 1 | 2.22 | LaboratoryFinding |
| IL_2 | 3 | 6.67 | LaboratoryFinding |
| IL_4 | 2 | 4.44 | LaboratoryFinding |
| IL_6 | 9 | 20.00 | LaboratoryFinding |
| IL_8 | 1 | 2.22 | LaboratoryFinding |
| Immune_enhancer | 2 | 4.44 | Treatment |
| Immune_suppressor | 1 | 2.22 | Treatment |
| Immunosuppression | 2 | 4.44 | Comorbidity/Complication |
| IMV_with_ECMO | 3 | 6.67 | Treatment |
| Interferon_inhalation | 3 | 6.67 | Treatment |
| International_normalized_ration | 1 | 2.22 | LaboratoryFinding |
| Intestinal_flora_regulating_drugs | 1 | 2.22 | Treatment |
| Intra_aortic_balloon_pump | 1 | 2.22 | Treatment |
| Intravenous_immunoglobulin | 11 | 24.44 | Treatment |
| Invasive_mechanical_ventilation | 13 | 28.89 | Treatment |
| Ischemia_modified_albumin | 1 | 2.22 | LaboratoryFinding |
| Lactate | 6 | 13.33 | LaboratoryFinding |
| Lactate_dehydrogenase | 25 | 55.56 | LaboratoryFinding |
| LDL | 1 | 2.22 | LaboratoryFinding |
| Leucocytes | 4 | 8.89 | LaboratoryFinding |
| Leukocytosis | 1 | 2.22 | LaboratoryFinding |
| Lithiasis | 1 | 2.22 | Comorbidity/Complication |
| Liver_disease | 7 | 15.56 | Comorbidity/Complication |
| Lopinavir_and_ritonavir | 4 | 8.89 | Treatment |
| Lung_disease | 2 | 4.44 | Comorbidity/Complication |
| Lung_severity_score | 1 | 2.22 | LaboratoryFinding |
| Lymphocytes | 36 | 80.00 | LaboratoryFinding |
| Lymphocytopenia | 1 | 2.22 | LaboratoryFinding |
| M_receptor_inhibitors | 1 | 2.22 | Treatment |
| Magnesium | 1 | 2.22 | LaboratoryFinding |
| Malaise | 2 | 4.44 | Symptom |
| MAP | 1 | 2.22 | LaboratoryFinding |
| Mechanical_ventilation | 7 | 15.56 | Treatment |
| Metabolic_Diseases | 1 | 2.22 | Comorbidity/Complication |
| Methylprednisolone | 2 | 4.44 | Treatment |
| Monocytes | 8 | 17.78 | LaboratoryFinding |
| Moxifloxacin_hydrochloride | 1 | 2.22 | Treatment |
| MuLBSTA | 1 | 2.22 | LaboratoryFinding |
| Multiple_Organ_Failure | 2 | 4.44 | Comorbidity/Complication |
| Myalgia_or_Fatigue | 33 | 73.33 | Symptom |
| Mycoplasma_pneumonia_antibody | 1 | 2.22 | LaboratoryFinding |
| Myoglobin | 6 | 13.33 | LaboratoryFinding |
| Nausea_or_Vomiting | 17 | 37.78 | Symptom |
| Nausea_or_vomiting | 1 | 2.22 | Symptom |
| Neutrophil_to_Lymphocyte_Ratio | 3 | 6.67 | LaboratoryFinding |
| Neutrophils | 29 | 64.44 | LaboratoryFinding |
| Nitrates | 1 | 2.22 | LaboratoryFinding |
| NK | 2 | 4.44 | LaboratoryFinding |
| No_symptoms | 1 | 2.22 | Symptom |
| No_comorbidity | 1 | 2.22 | Comorbidity/Complication |
| Non_invasive_mechanical_ventilation | 13 | 28.89 | Treatment |
| NT_proBNP | 4 | 8.89 | LaboratoryFinding |
| Obesity | 1 | 2.22 | Comorbidity/Complication |
| Oseltamivir | 4 | 8.89 | Treatment |
| Osteoarticular_surgery | 1 | 2.22 | Comorbidity/Complication |
| Other_disease | 5 | 11.11 | Comorbidity/Complication |
| Other_diseases | 1 | 2.22 | Comorbidity/Complication |
| Other_treatment | 1 | 2.22 | Treatment |
| Oxygen_Inhalation_Therapy | 17 | 37.78 | Treatment |
| Oxygen_saturation | 4 | 8.89 | LaboratoryFinding |
| Oxygenation_index | 2 | 4.44 | LaboratoryFinding |
| PaCO2 | 2 | 4.44 | LaboratoryFinding |
| PaO2 | 1 | 2.22 | LaboratoryFinding |
| PaO2_FiO2_ratio | 5 | 11.11 | LaboratoryFinding |
| pH | 1 | 2.22 | LaboratoryFinding |
| Pharyngeal_discomfort | 1 | 2.22 | LaboratoryFinding |
| Phosphorus | 1 | 2.22 | LaboratoryFinding |
| Platelets | 32 | 71.11 | LaboratoryFinding |
| Pneumonia | 3 | 6.67 | Comorbidity/Complication |
| Pneumothorax | 3 | 6.67 | Comorbidity/Complication |
| Potassium | 11 | 24.44 | LaboratoryFinding |
| Prealbumin | 4 | 8.89 | LaboratoryFinding |
| Procacitonin | 3 | 6.67 | LaboratoryFinding |
| Procalcitonin | 22 | 48.89 | LaboratoryFinding |
| Prothrombin_time | 17 | 37.78 | LaboratoryFinding |
| Pseudoepinephrine | 1 | 2.22 | Treatment |
| Pulmonary_Tuberculosis | 1 | 2.22 | Comorbidity/Complication |
| Pulse_rate | 1 | 2.22 | LaboratoryFinding |
| Rash | 1 | 2.22 | Symptom |
| Recombinant_Cytokine_Gene_Derived_Protein | 1 | 2.22 | Treatment |
| Red_blood_cell | 1 | 2.22 | LaboratoryFinding |
| Reduling | 1 | 2.22 | Treatment |
| Renal_Insufficiency | 1 | 2.22 | Comorbidity/Complication |
| Renal_replacement_therapy | 9 | 20.00 | Treatment |
| Respiratory_failure_and_disease | 6 | 13.33 | Comorbidity/Complication |
| Rhabdomyolysis | 1 | 2.22 | Comorbidity/Complication |
| Ribavirin | 1 | 2.22 | Treatment |
| RR | 11 | 24.44 | LaboratoryFinding |
| Schizophrenia | 1 | 2.22 | Comorbidity/Complication |
| Sepsis | 2 | 4.44 | Comorbidity/Complication |
| Septic_shock | 4 | 8.89 | Comorbidity/Complication |
| Serum_amyloid_A | 2 | 4.44 | LaboratoryFinding |
| Serum_ferritin | 6 | 13.33 | LaboratoryFinding |
| Sex | 36 | 80.00 | Demographic |
| Shock | 6 | 13.33 | Comorbidity/Complication |
| Sinusitis | 1 | 2.22 | Comorbidity/Complication |
| Smoking | 16 | 35.56 | Demographic |
| Sodium | 10 | 22.22 | LaboratoryFinding |
| SOFA_score | 5 | 11.11 | LaboratoryFinding |
| Sputum | 20 | 44.44 | Symptom |
| Stroke | 2 | 4.44 | Comorbidity/Complication |
| Stuffy_or_Runny_nose | 15 | 33.33 | Symptom |
| Surgery_history | 1 | 2.22 | Comorbidity/Complication |
| Systolic_blood_pressure | 9 | 20.00 | LaboratoryFinding |
| T_Lymphocytes | 3 | 6.67 | LaboratoryFinding |
| Temperature | 13 | 28.89 | LaboratoryFinding |
| Thrombocytopenia | 2 | 4.44 | LaboratoryFinding |
| Thymalfasin | 1 | 2.22 | Treatment |
| Thymopentin | 1 | 2.22 | Treatment |
| Thyroid_Disease | 3 | 6.67 | Comorbidity/Complication |
| TNF | 3 | 6.67 | LaboratoryFinding |
| Tonsil_swelling | 2 | 4.44 | Symptom |
| Total_bilirubin | 18 | 40.00 | LaboratoryFinding |
| Total_cholesterol | 1 | 2.22 | LaboratoryFinding |
| Total_protein | 2 | 4.44 | LaboratoryFinding |
| Total_radiograph_score | 1 | 2.22 | LaboratoryFinding |
| Trachea_cannula | 2 | 4.44 | Treatment |
| Tracheotomy | 1 | 2.22 | Treatment |
| Treg | 1 | 2.22 | LaboratoryFinding |
| Triglyceride | 1 | 2.22 | LaboratoryFinding |
| Tuberculosis | 2 | 4.44 | Comorbidity/Complication |
| Urea | 2 | 4.44 | LaboratoryFinding |
| Urea_nitrogen | 17 | 37.78 | LaboratoryFinding |
| Ureterotomy | 1 | 2.22 | Comorbidity/Complication |
| Uric_acid | 1 | 2.22 | LaboratoryFinding |
| Urinary_tract_infection | 1 | 2.22 | Comorbidity/Complication |
| Urine_protein | 1 | 2.22 | LaboratoryFinding |
| Urolithiasis | 1 | 2.22 | Comorbidity/Complication |
| Urticaria | 1 | 2.22 | Comorbidity/Complication |
| Varicose_vein_surgery | 1 | 2.22 | Comorbidity/Complication |
| Vasoconstrictive_agents | 1 | 2.22 | Treatment |
| Vasopressors | 2 | 4.44 | Comorbidity/Complication |
| Viral_myocarditis | 1 | 2.22 | Comorbidity/Complication |
| Weakness | 2 | 4.44 | Symptom |
| White_blood_cell | 31 | 68.89 | LaboratoryFinding |
| Xuebijing | 1 | 2.22 | Treatment |
| α-HBDH | 2 | 4.44 | LaboratoryFinding |
| β blocker | 1 | 2.22 | Treatment |

**Table S3. Association between clinical and demographic variables and outcomes of COVID-19**

|  |  |  | **Association type (%)** | | |
| --- | --- | --- | --- | --- | --- |
| **Variable** | **num_study** | **class** | **Positive** | **No** | **Negative** |
| Abdominal pain | 7 | Symptom | 85.71 | 14.29 | 0.00 |
| Acid suppression drugs | 1 | Treatment | 100.00 | 0.00 | 0.00 |
| Acidosis | 1 | Comorbidity/Complication | 100.00 | 0.00 | 0.00 |
| Activated partial thromboplastin time | 8 | LaboratoryFinding | 12.50 | 75.00 | 12.50 |
| Acute cardiac injury | 8 | Comorbidity/Complication | 87.50 | 12.50 | 0.00 |
| Acute kidney injury | 12 | Comorbidity/Complication | 83.33 | 16.67 | 0.00 |
| Acute liver injury | 2 | Comorbidity/Complication | 50.00 | 50.00 | 0.00 |
| Acute lung injury | 1 | Comorbidity/Complication | 100.00 | 0.00 | 0.00 |
| Acute respiratory distress syndrome | 14 | Comorbidity/Complication | 85.71 | 14.29 | 0.00 |
| Age | 42 | Age | 76.19 | 16.67 | 7.14 |
| Alanine aminotransferase | 33 | LaboratoryFinding | 39.39 | 60.61 | 0.00 |
| Albumin | 25 | LaboratoryFinding | 12.00 | 36.00 | 52.00 |
| Albumin-Globulin ratio | 1 | LaboratoryFinding | 0.00 | 100.00 | 0.00 |
| Alcoholic hepatitis | 1 | Comorbidity/Complication | 100.00 | 0.00 | 0.00 |
| Allergic rhinitis | 1 | Symptom | 100.00 | 0.00 | 0.00 |
| Anemia | 3 | Symptom | 100.00 | 0.00 | 0.00 |
| Anorexia | 15 | Symptom | 66.67 | 20.00 | 13.33 |
| Antibiotic therapy | 19 | Treatment | 31.58 | 63.16 | 5.26 |
| Antifungal therapy | 8 | Treatment | 100.00 | 0.00 | 0.00 |
| Antiviral therapy | 19 | Treatment | 5.26 | 73.68 | 21.05 |
| Aortic sclerosis | 1 | Comorbidity/Complication | 0.00 | 100.00 | 0.00 |
| APACHE II | 5 | LaboratoryFinding | 80.00 | 20.00 | 0.00 |
| Appendectomy | 1 | Comorbidity/Complication | 100.00 | 0.00 | 0.00 |
| APTT | 6 | LaboratoryFinding | 16.67 | 66.67 | 16.67 |
| Arbidol | 5 | Treatment | 0.00 | 40.00 | 60.00 |
| Arrhythmia | 6 | Comorbidity/Complication | 83.33 | 16.67 | 0.00 |
| Arterial pressure | 3 | LaboratoryFinding | 0.00 | 100.00 | 0.00 |
| Arthralgia | 2 | Symptom | 0.00 | 0.00 | 100.00 |
| Aspartate aminotransferase | 33 | LaboratoryFinding | 66.67 | 33.33 | 0.00 |
| Aspartate aminotransferase / Alanine aminotransferase ratio | 1 | LaboratoryFinding | 100.00 | 0.00 | 0.00 |
| Asthma | 2 | Comorbidity/Complication | 50.00 | 0.00 | 50.00 |
| Atopic dermatitis | 1 | Symptom | 100.00 | 0.00 | 0.00 |
| Atrial fibrillation | 1 | Comorbidity/Complication | 0.00 | 100.00 | 0.00 |
| Autoimmune Diseases or Immunodeficiency | 3 | Comorbidity/Complication | 33.33 | 0.00 | 66.67 |
| B Lymphocytes | 5 | LaboratoryFinding | 20.00 | 60.00 | 20.00 |
| B-type natriuretic peptide | 2 | LaboratoryFinding | 100.00 | 0.00 | 0.00 |
| Bacteraemia | 1 | Comorbidity/Complication | 100.00 | 0.00 | 0.00 |
| Bacterial pneumonia | 1 | Comorbidity/Complication | 100.00 | 0.00 | 0.00 |
| Barotrauma | 1 | Comorbidity/Complication | 100.00 | 0.00 | 0.00 |
| Basophils | 2 | LaboratoryFinding | 0.00 | 0.00 | 100.00 |
| Blood Coagulation Disorders | 2 | Comorbidity/Complication | 50.00 | 0.00 | 50.00 |
| Blood glucose | 2 | LaboratoryFinding | 0.00 | 100.00 | 0.00 |
| BMI | 2 | LaboratoryFinding | 0.00 | 100.00 | 0.00 |
| Brain disease | 1 | Comorbidity/Complication | 100.00 | 0.00 | 0.00 |
| C-reactive protein | 32 | LaboratoryFinding | 84.38 | 12.50 | 3.13 |
| Ca2+-receptor blocker | 1 | Treatment | 100.00 | 0.00 | 0.00 |
| Calcium | 4 | LaboratoryFinding | 0.00 | 25.00 | 75.00 |
| Cancer | 19 | Comorbidity/Complication | 57.89 | 15.79 | 26.32 |
| Cardiac disease and injury | 7 | Comorbidity/Complication | 57.14 | 28.57 | 14.29 |
| Cardiac intervention | 1 | Comorbidity/Complication | 100.00 | 0.00 | 0.00 |
| Cardiac troponin | 17 | LaboratoryFinding | 82.35 | 17.65 | 0.00 |
| Cardio-pulmonary resuscitation | 1 | Treatment | 100.00 | 0.00 | 0.00 |
| Cardiopathy | 1 | Comorbidity/Complication | 100.00 | 0.00 | 0.00 |
| Cardiovascular disease | 21 | Comorbidity/Complication | 85.71 | 9.52 | 4.76 |
| CD28+CD4+T cells/CD4+T | 1 | LaboratoryFinding | 0.00 | 100.00 | 0.00 |
| CD28+CD8+T | 1 | LaboratoryFinding | 0.00 | 100.00 | 0.00 |
| CD3 | 7 | LaboratoryFinding | 14.29 | 14.29 | 71.43 |
| CD4 | 11 | LaboratoryFinding | 9.09 | 27.27 | 63.64 |
| CD4/8 | 1 | LaboratoryFinding | 0.00 | 100.00 | 0.00 |
| CD45 | 1 | LaboratoryFinding | 0.00 | 0.00 | 100.00 |
| CD45RA+ Treg | 1 | LaboratoryFinding | 0.00 | 0.00 | 100.00 |
| CD45RA+CD4+T cells/ CD4+T | 1 | LaboratoryFinding | 0.00 | 100.00 | 0.00 |
| CD45RO+CD4+T cells/ CD4+T | 1 | LaboratoryFinding | 0.00 | 100.00 | 0.00 |
| CD45RO+Treg | 1 | LaboratoryFinding | 0.00 | 100.00 | 0.00 |
| CD8 | 11 | LaboratoryFinding | 9.09 | 27.27 | 63.64 |
| Cerebrovascular disease | 13 | Comorbidity/Complication | 69.23 | 7.69 | 23.08 |
| Cesarean section | 1 | Comorbidity/Complication | 0.00 | 0.00 | 100.00 |
| Chest congestion | 1 | Symptom | 100.00 | 0.00 | 0.00 |
| Chest pain | 8 | Symptom | 25.00 | 25.00 | 50.00 |
| Chest tightness or Dyspnea | 48 | Symptom | 68.75 | 20.83 | 10.42 |
| Chills | 7 | Symptom | 57.14 | 42.86 | 0.00 |
| Chinese Medicine | 5 | Treatment | 40.00 | 60.00 | 0.00 |
| Chlamydia pneumonia antibody | 2 | LaboratoryFinding | 0.00 | 0.00 | 100.00 |
| Chloride | 5 | LaboratoryFinding | 0.00 | 80.00 | 20.00 |
| Chloroquine or Hydroxychloroquine | 2 | Treatment | 50.00 | 50.00 | 0.00 |
| Cholecystectomy | 1 | Comorbidity/Complication | 100.00 | 0.00 | 0.00 |
| Cholelithiasis | 1 | Comorbidity/Complication | 100.00 | 0.00 | 0.00 |
| Cholinesterase | 2 | LaboratoryFinding | 0.00 | 0.00 | 100.00 |
| Chronic gastritis and gastric ulcer | 1 | Comorbidity/Complication | 0.00 | 0.00 | 100.00 |
| Chronic hepatitis | 3 | Comorbidity/Complication | 66.67 | 0.00 | 33.33 |
| Chronic kidney disease | 15 | Comorbidity/Complication | 93.33 | 0.00 | 6.67 |
| Chronic liver disease | 9 | Comorbidity/Complication | 44.44 | 11.11 | 44.44 |
| Chronic obstructive lung disease | 16 | Comorbidity/Complication | 87.50 | 12.50 | 0.00 |
| Chronic pharyngitis | 1 | Comorbidity/Complication | 0.00 | 0.00 | 100.00 |
| Chronic pulmonary disease | 2 | Comorbidity/Complication | 50.00 | 0.00 | 50.00 |
| Chronic Respiratory Disease | 1 | Comorbidity/Complication | 0.00 | 100.00 | 0.00 |
| CK-MB | 12 | LaboratoryFinding | 33.33 | 66.67 | 0.00 |
| Co-infection of Bacteria | 3 | Comorbidity/Complication | 100.00 | 0.00 | 0.00 |
| Co-infection of Fungus | 5 | Comorbidity/Complication | 100.00 | 0.00 | 0.00 |
| Co-infection of Other viruses | 11 | Comorbidity/Complication | 72.73 | 18.18 | 9.09 |
| Coma | 1 | Symptom | 100.00 | 0.00 | 0.00 |
| Combined treatment | 1 | Treatment | 100.00 | 0.00 | 0.00 |
| Comorbidity | 17 | Comorbidity/Complication | 94.12 | 5.88 | 0.00 |
| Complement 3 | 3 | LaboratoryFinding | 33.33 | 66.67 | 0.00 |
| Complement 4 | 3 | LaboratoryFinding | 0.00 | 100.00 | 0.00 |
| Confusion or Fluster | 2 | Symptom | 100.00 | 0.00 | 0.00 |
| Conjunctival congestion | 2 | Symptom | 50.00 | 0.00 | 50.00 |
| Convalescent plasma | 1 | Treatment | 0.00 | 100.00 | 0.00 |
| Corticosteroid | 7 | Treatment | 85.71 | 14.29 | 0.00 |
| Cough | 43 | Symptom | 16.28 | 79.07 | 4.65 |
| Craniocerebral surgery | 1 | Comorbidity/Complication | 0.00 | 100.00 | 0.00 |
| Creatine kinase | 29 | LaboratoryFinding | 44.83 | 48.28 | 6.90 |
| Creatinine | 34 | LaboratoryFinding | 47.06 | 52.94 | 0.00 |
| creatinine | 2 | LaboratoryFinding | 0.00 | 100.00 | 0.00 |
| Cumulative fluid balance in ICU | 2 | Treatment | 100.00 | 0.00 | 0.00 |
| CURB-65 score | 3 | LaboratoryFinding | 100.00 | 0.00 | 0.00 |
| Cystatin C | 2 | LaboratoryFinding | 100.00 | 0.00 | 0.00 |
| D-dimer | 28 | LaboratoryFinding | 89.29 | 10.71 | 0.00 |
| Diabetes | 33 | Comorbidity/Complication | 69.70 | 27.27 | 3.03 |
| Diarrhea | 31 | Symptom | 41.94 | 35.48 | 22.58 |
| Diastolic blood pressure | 4 | LaboratoryFinding | 0.00 | 100.00 | 0.00 |
| Digestive tracthemorrhage | 1 | Comorbidity/Complication | 100.00 | 0.00 | 0.00 |
| Direct Bilirubin | 2 | LaboratoryFinding | 50.00 | 50.00 | 0.00 |
| Disseminated intravascular coagulation | 4 | Comorbidity/Complication | 100.00 | 0.00 | 0.00 |
| Diuretics | 1 | Treatment | 0.00 | 100.00 | 0.00 |
| Drinking | 3 | Drinking | 0.00 | 66.67 | 33.33 |
| Drug hypersensitivity | 1 | Symptom | 0.00 | 100.00 | 0.00 |
| Dry or Soar throat | 26 | Symptom | 26.92 | 38.46 | 34.62 |
| Dysbiosis | 1 | Comorbidity/Complication | 0.00 | 100.00 | 0.00 |
| ECMO | 9 | Treatment | 88.89 | 11.11 | 0.00 |
| Electrolyte imbalance | 3 | Comorbidity/Complication | 100.00 | 0.00 | 0.00 |
| Enlargement of lymph nodes or sinus | 3 | Symptom | 100.00 | 0.00 | 0.00 |
| Eosinophils | 10 | LaboratoryFinding | 10.00 | 0.00 | 90.00 |
| Epilepsy | 1 | Comorbidity/Complication | 100.00 | 0.00 | 0.00 |
| Eructation | 1 | Symptom | 0.00 | 100.00 | 0.00 |
| Erythrocyte sedimentation rate | 10 | LaboratoryFinding | 40.00 | 60.00 | 0.00 |
| Estimated Glomerular Filtration Rate | 2 | LaboratoryFinding | 0.00 | 0.00 | 100.00 |
| Expectorant or Antitussive drugs | 2 | Treatment | 100.00 | 0.00 | 0.00 |
| Fever | 42 | Symptom | 23.81 | 69.05 | 7.14 |
| Fibrinogen | 7 | LaboratoryFinding | 28.57 | 71.43 | 0.00 |
| Food allergy | 1 | Symptom | 100.00 | 0.00 | 0.00 |
| Gastric mucosa repair agent | 1 | Treatment | 100.00 | 0.00 | 0.00 |
| Gastrointestinal disorder | 1 | Comorbidity/Complication | 100.00 | 0.00 | 0.00 |
| Gastrointestinal haemorrhage | 2 | Comorbidity/Complication | 100.00 | 0.00 | 0.00 |
| Gastrointestinal symptoms | 1 | Symptom | 0.00 | 100.00 | 0.00 |
| Globulin | 4 | LaboratoryFinding | 25.00 | 50.00 | 25.00 |
| Glucocorticoid therapy | 15 | Treatment | 46.67 | 46.67 | 6.67 |
| Glucose | 5 | LaboratoryFinding | 40.00 | 60.00 | 0.00 |
| Glutathione reductase | 1 | LaboratoryFinding | 100.00 | 0.00 | 0.00 |
| Glycosylated hemoglobin | 1 | LaboratoryFinding | 100.00 | 0.00 | 0.00 |
| HCO3- | 1 | LaboratoryFinding | 0.00 | 100.00 | 0.00 |
| Headache or Dizziness | 35 | Symptom | 31.43 | 34.29 | 34.29 |
| Heart disease | 1 | Comorbidity/Complication | 100.00 | 0.00 | 0.00 |
| Heart Failure | 3 | Comorbidity/Complication | 100.00 | 0.00 | 0.00 |
| Hemoglobin | 23 | LaboratoryFinding | 4.35 | 78.26 | 17.39 |
| Hemoptysis | 7 | Symptom | 85.71 | 14.29 | 0.00 |
| Hemorrhoidectomy | 1 | Comorbidity/Complication | 100.00 | 0.00 | 0.00 |
| Heparin | 1 | Treatment | 0.00 | 100.00 | 0.00 |
| Hepatic abnormality | 1 | Comorbidity/Complication | 100.00 | 0.00 | 0.00 |
| Hepatitis B infection | 1 | Comorbidity/Complication | 0.00 | 0.00 | 100.00 |
| HIV infection | 2 | Comorbidity/Complication | 50.00 | 0.00 | 50.00 |
| HLA-DR+CD8+T cells/ CD8+T | 1 | LaboratoryFinding | 0.00 | 100.00 | 0.00 |
| Hormone Therapy | 1 | Treatment | 100.00 | 0.00 | 0.00 |
| HR | 11 | LaboratoryFinding | 36.36 | 54.55 | 9.09 |
| Hyperlipidemia | 2 | Comorbidity/Complication | 0.00 | 0.00 | 100.00 |
| Hypertension | 33 | Comorbidity/Complication | 75.76 | 24.24 | 0.00 |
| Hypoalbuminemia | 1 | Comorbidity/Complication | 100.00 | 0.00 | 0.00 |
| Hypoproteinemia | 2 | Comorbidity/Complication | 100.00 | 0.00 | 0.00 |
| Hysterectomy | 1 | Comorbidity/Complication | 100.00 | 0.00 | 0.00 |
| IFN-Œ≥ | 1 | LaboratoryFinding | 0.00 | 0.00 | 100.00 |
| IGA | 1 | LaboratoryFinding | 0.00 | 100.00 | 0.00 |
| IGG | 1 | LaboratoryFinding | 0.00 | 100.00 | 0.00 |
| IGM | 1 | LaboratoryFinding | 0.00 | 100.00 | 0.00 |
| IL-1 | 1 | LaboratoryFinding | 100.00 | 0.00 | 0.00 |
| IL-10 | 3 | LaboratoryFinding | 100.00 | 0.00 | 0.00 |
| IL-17A | 1 | LaboratoryFinding | 100.00 | 0.00 | 0.00 |
| IL-2 | 3 | LaboratoryFinding | 33.33 | 66.67 | 0.00 |
| IL-4 | 2 | LaboratoryFinding | 0.00 | 100.00 | 0.00 |
| IL-6 | 10 | LaboratoryFinding | 100.00 | 0.00 | 0.00 |
| IL-8 | 1 | LaboratoryFinding | 100.00 | 0.00 | 0.00 |
| Immune enhancer | 2 | Treatment | 100.00 | 0.00 | 0.00 |
| Immune suppressor | 1 | Treatment | 100.00 | 0.00 | 0.00 |
| Immunosuppression | 2 | Comorbidity/Complication | 0.00 | 50.00 | 50.00 |
| IMV with ECMO | 4 | Treatment | 100.00 | 0.00 | 0.00 |
| Interferon inhalation | 3 | Treatment | 33.33 | 33.33 | 33.33 |
| International normalized ration | 2 | LaboratoryFinding | 50.00 | 50.00 | 0.00 |
| Intestinal flora regulating drugs | 1 | Treatment | 0.00 | 100.00 | 0.00 |
| Intra-aortic balloon pump | 1 | Treatment | 0.00 | 0.00 | 100.00 |
| Intravenous immunoglobulin | 13 | Treatment | 61.54 | 30.77 | 7.69 |
| Invasive mechanical ventilation | 15 | Treatment | 93.33 | 6.67 | 0.00 |
| Ischemia-modified albumin | 1 | LaboratoryFinding | 100.00 | 0.00 | 0.00 |
| Lactate | 7 | LaboratoryFinding | 57.14 | 42.86 | 0.00 |
| Lactate dehydrogenase | 29 | LaboratoryFinding | 82.76 | 13.79 | 3.45 |
| LDL | 2 | LaboratoryFinding | 0.00 | 50.00 | 50.00 |
| Leucocytes | 5 | LaboratoryFinding | 60.00 | 20.00 | 20.00 |
| Leukocytosis | 1 | LaboratoryFinding | 100.00 | 0.00 | 0.00 |
| Lithiasis | 1 | Comorbidity/Complication | 100.00 | 0.00 | 0.00 |
| Liver disease | 7 | Comorbidity/Complication | 57.14 | 28.57 | 14.29 |
| Lopinavir and ritonavir | 4 | Treatment | 50.00 | 50.00 | 0.00 |
| Lung disease | 2 | Comorbidity/Complication | 100.00 | 0.00 | 0.00 |
| Lung severity score | 1 | LaboratoryFinding | 0.00 | 100.00 | 0.00 |
| Lymphocytes | 46 | LaboratoryFinding | 0.00 | 26.09 | 73.91 |
| Lymphocytopenia | 1 | LaboratoryFinding | 0.00 | 0.00 | 100.00 |
| M-receptor inhibitors | 1 | Treatment | 100.00 | 0.00 | 0.00 |
| Magnesium | 1 | LaboratoryFinding | 0.00 | 100.00 | 0.00 |
| Malaise | 2 | Symptom | 100.00 | 0.00 | 0.00 |
| MAP | 1 | LaboratoryFinding | 0.00 | 100.00 | 0.00 |
| Mechanical ventilation | 9 | Treatment | 77.78 | 22.22 | 0.00 |
| Metabolic Diseases | 1 | Comorbidity/Complication | 100.00 | 0.00 | 0.00 |
| Methylprednisolone | 3 | Treatment | 33.33 | 66.67 | 0.00 |
| Monocytes | 9 | LaboratoryFinding | 0.00 | 77.78 | 22.22 |
| Moxifloxacin hydrochloride | 1 | Treatment | 0.00 | 100.00 | 0.00 |
| MuLBSTA | 1 | LaboratoryFinding | 100.00 | 0.00 | 0.00 |
| Multiple Organ Failure | 2 | Comorbidity/Complication | 100.00 | 0.00 | 0.00 |
| Myalgia or Fatigue | 55 | Symptom | 25.45 | 61.82 | 12.73 |
| Mycoplasma pneumonia antibody | 2 | LaboratoryFinding | 0.00 | 50.00 | 50.00 |
| Myoglobin | 6 | LaboratoryFinding | 100.00 | 0.00 | 0.00 |
| Nausea or Vomiting | 24 | Symptom | 45.83 | 20.83 | 33.33 |
| Nausea or vomiting | 1 | Symptom | 0.00 | 0.00 | 100.00 |
| Neutrophil-to-Lymphocyte Ratio | 3 | LaboratoryFinding | 66.67 | 33.33 | 0.00 |
| Neutrophils | 34 | LaboratoryFinding | 61.76 | 32.35 | 5.88 |
| Nitrates | 1 | LaboratoryFinding | 100.00 | 0.00 | 0.00 |
| NK | 5 | LaboratoryFinding | 0.00 | 80.00 | 20.00 |
| No symptoms | 1 | Symptom | 0.00 | 100.00 | 0.00 |
| No-comorbidity | 1 | Comorbidity/Complication | 0.00 | 100.00 | 0.00 |
| Non-invasive mechanical ventilation | 15 | Treatment | 93.33 | 0.00 | 6.67 |
| NT-proBNP | 4 | LaboratoryFinding | 50.00 | 50.00 | 0.00 |
| Obesity | 1 | Comorbidity/Complication | 100.00 | 0.00 | 0.00 |
| Oseltamivir | 5 | Treatment | 60.00 | 20.00 | 20.00 |
| Osteoarticular surgery | 1 | Comorbidity/Complication | 100.00 | 0.00 | 0.00 |
| Other disease | 5 | Comorbidity/Complication | 100.00 | 0.00 | 0.00 |
| Other diseases | 1 | Comorbidity/Complication | 100.00 | 0.00 | 0.00 |
| Other treatment | 1 | Treatment | 0.00 | 100.00 | 0.00 |
| Oxygen Inhalation Therapy | 20 | Treatment | 65.00 | 5.00 | 30.00 |
| Oxygen saturation | 4 | LaboratoryFinding | 0.00 | 75.00 | 25.00 |
| Oxygenation index | 2 | LaboratoryFinding | 50.00 | 0.00 | 50.00 |
| PaCO2 | 2 | LaboratoryFinding | 0.00 | 100.00 | 0.00 |
| PaO2 | 1 | LaboratoryFinding | 0.00 | 100.00 | 0.00 |
| PaO2/FiO2 ratio | 7 | LaboratoryFinding | 0.00 | 28.57 | 71.43 |
| pH | 1 | LaboratoryFinding | 0.00 | 0.00 | 100.00 |
| Pharyngeal discomfort | 2 | LaboratoryFinding | 0.00 | 0.00 | 100.00 |
| Phosphorus | 1 | LaboratoryFinding | 0.00 | 100.00 | 0.00 |
| Platelets | 34 | LaboratoryFinding | 0.00 | 64.71 | 35.29 |
| Pneumonia | 4 | Comorbidity/Complication | 25.00 | 50.00 | 25.00 |
| Pneumothorax | 3 | Comorbidity/Complication | 66.67 | 0.00 | 33.33 |
| Potassium | 12 | LaboratoryFinding | 8.33 | 91.67 | 0.00 |
| Prealbumin | 5 | LaboratoryFinding | 0.00 | 20.00 | 80.00 |
| Procacitonin | 3 | LaboratoryFinding | 66.67 | 0.00 | 33.33 |
| Procalcitonin | 24 | LaboratoryFinding | 62.50 | 33.33 | 4.17 |
| Prothrombin time | 18 | LaboratoryFinding | 33.33 | 66.67 | 0.00 |
| Pseudoepinephrine | 1 | Treatment | 100.00 | 0.00 | 0.00 |
| Pulmonary_Tuberculosis | 1 | Comorbidity/Complication | 100.00 | 0.00 | 0.00 |
| Pulse rate | 1 | LaboratoryFinding | 0.00 | 100.00 | 0.00 |
| Rash | 2 | Symptom | 50.00 | 0.00 | 50.00 |
| Recombinant Cytokine Gene Derived Protein | 1 | Treatment | 100.00 | 0.00 | 0.00 |
| Red blood cell | 1 | LaboratoryFinding | 0.00 | 100.00 | 0.00 |
| Reduling | 1 | Treatment | 0.00 | 100.00 | 0.00 |
| Renal Insufficiency | 2 | Comorbidity/Complication | 50.00 | 0.00 | 50.00 |
| Renal replacement therapy | 10 | Treatment | 90.00 | 10.00 | 0.00 |
| Respiratory failure and disease | 8 | Comorbidity/Complication | 62.50 | 12.50 | 25.00 |
| Rhabdomyolysis | 2 | Comorbidity/Complication | 0.00 | 0.00 | 100.00 |
| Ribavirin | 1 | Treatment | 0.00 | 100.00 | 0.00 |
| RR | 11 | LaboratoryFinding | 45.45 | 54.55 | 0.00 |
| Schizophrenia | 1 | Comorbidity/Complication | 0.00 | 0.00 | 100.00 |
| Sepsis | 2 | Comorbidity/Complication | 100.00 | 0.00 | 0.00 |
| Septic_shock | 5 | Comorbidity/Complication | 100.00 | 0.00 | 0.00 |
| Serum amyloid A | 2 | LaboratoryFinding | 50.00 | 50.00 | 0.00 |
| Serum ferritin | 7 | LaboratoryFinding | 71.43 | 28.57 | 0.00 |
| Sex | 40 | Sex | 67.50 | 32.50 | 0.00 |
| Shock | 6 | Comorbidity/Complication | 100.00 | 0.00 | 0.00 |
| Sinusitis | 1 | Comorbidity/Complication | 0.00 | 0.00 | 100.00 |
| Smoking | 17 | Smoker | 35.29 | 41.18 | 23.53 |
| Sodium | 12 | LaboratoryFinding | 0.00 | 50.00 | 50.00 |
| SOFA score | 7 | LaboratoryFinding | 85.71 | 14.29 | 0.00 |
| Sputum | 22 | Symptom | 22.73 | 77.27 | 0.00 |
| Stroke | 2 | Comorbidity/Complication | 100.00 | 0.00 | 0.00 |
| Stuffy or Runny nose | 18 | Symptom | 22.22 | 27.78 | 50.00 |
| Surgery history | 1 | Comorbidity/Complication | 0.00 | 100.00 | 0.00 |
| Systolic blood pressure | 9 | LaboratoryFinding | 22.22 | 66.67 | 11.11 |
| T Lymphocytes | 11 | LaboratoryFinding | 0.00 | 54.55 | 45.45 |
| Temperature | 18 | LaboratoryFinding | 27.78 | 66.67 | 5.56 |
| Thrombocytopenia | 2 | LaboratoryFinding | 50.00 | 50.00 | 0.00 |
| Thymalfasin | 1 | Treatment | 100.00 | 0.00 | 0.00 |
| Thymopentin | 1 | Treatment | 0.00 | 100.00 | 0.00 |
| Thyroid Disease | 3 | Comorbidity/Complication | 100.00 | 0.00 | 0.00 |
| TNF | 4 | LaboratoryFinding | 50.00 | 50.00 | 0.00 |
| Tonsil swelling | 3 | Symptom | 66.67 | 33.33 | 0.00 |
| Total bilirubin | 22 | LaboratoryFinding | 54.55 | 36.36 | 9.09 |
| Total cholesterol | 1 | LaboratoryFinding | 0.00 | 100.00 | 0.00 |
| Total protein | 3 | LaboratoryFinding | 0.00 | 66.67 | 33.33 |
| Total radiograph score | 1 | LaboratoryFinding | 100.00 | 0.00 | 0.00 |
| Trachea cannula | 2 | Treatment | 100.00 | 0.00 | 0.00 |
| Tracheotomy | 1 | Treatment | 100.00 | 0.00 | 0.00 |
| Treg | 1 | LaboratoryFinding | 0.00 | 100.00 | 0.00 |
| Triglyceride | 1 | LaboratoryFinding | 0.00 | 100.00 | 0.00 |
| Tuberculosis | 2 | Comorbidity/Complication | 100.00 | 0.00 | 0.00 |
| Urea | 3 | LaboratoryFinding | 100.00 | 0.00 | 0.00 |
| Urea nitrogen | 19 | LaboratoryFinding | 52.63 | 47.37 | 0.00 |
| Ureterotomy | 1 | Comorbidity/Complication | 100.00 | 0.00 | 0.00 |
| Uric acid | 1 | LaboratoryFinding | 100.00 | 0.00 | 0.00 |
| Urinary tract infection | 1 | Comorbidity/Complication | 100.00 | 0.00 | 0.00 |
| Urine protein | 1 | LaboratoryFinding | 0.00 | 0.00 | 100.00 |
| Urolithiasis | 1 | Comorbidity/Complication | 0.00 | 100.00 | 0.00 |
| Urticaria | 1 | Comorbidity/Complication | 0.00 | 100.00 | 0.00 |
| Varicose vein surgery | 1 | Comorbidity/Complication | 100.00 | 0.00 | 0.00 |
| Vasoconstrictive agents | 1 | Treatment | 100.00 | 0.00 | 0.00 |
| Vasopressors | 2 | Comorbidity/Complication | 100.00 | 0.00 | 0.00 |
| Viral myocarditis | 1 | Comorbidity/Complication | 100.00 | 0.00 | 0.00 |
| Weakness | 2 | Symptom | 50.00 | 50.00 | 0.00 |
| White blood cell | 35 | LaboratoryFinding | 45.71 | 54.29 | 0.00 |
| Xuebijing | 1 | Treatment | 100.00 | 0.00 | 0.00 |
| Œ±-HBDH | 3 | LaboratoryFinding | 100.00 | 0.00 | 0.00 |
| Œ≤-blocker | 1 | Treatment | 0.00 | 0.00 | 100.00 |

**Table S4. List of clinical and demographic variables in biomedical literature**

|  | Fraction of positive associations (%) | | | | | | |
| --- | --- | --- | --- | --- | --- | --- | --- |
| Variable | ARDS | Severity | Death | ICU | Diagnosis | Hospitalization | O2 saturation |
| Abdominal pain | 0.00 | 75.00 | 0.00 | 0.00 | 0.00 | 0.00 | 0.00 |
| Activated partial thromboplastin time | 0.00 | 0.00 | 0.00 | 33.33 | 0.00 | 0.00 | 0.00 |
| Acute cardiac injury | 0.00 | 100.00 | 50.00 | 100.00 | 0.00 | 0.00 | 0.00 |
| Acute kidney injury | 0.00 | 100.00 | 66.67 | 100.00 | 0.00 | 0.00 | 0.00 |
| Acute respiratory distress syndrome | 0.00 | 100.00 | 100.00 | 100.00 | 0.00 | 0.00 | 0.00 |
| Age | 100.00 | 90.00 | 66.67 | 66.67 | 0.00 | 50.00 | 100.00 |
| Alanine aminotransferase | 50.00 | 31.25 | 60.00 | 100.00 | 0.00 | 0.00 | 0.00 |
| Albumin | 0.00 | 15.38 | 0.00 | 0.00 | 0.00 | 0.00 | 0.00 |
| Anemia | 0.00 | 0.00 | 100.00 | 0.00 | 0.00 | 0.00 | 0.00 |
| Anorexia | 0.00 | 80.00 | 0.00 | 0.00 | 0.00 | 0.00 | 0.00 |
| Antibiotic therapy | 0.00 | 66.67 | 0.00 | 0.00 | 0.00 | 0.00 | 0.00 |
| Antifungal therapy | 0.00 | 100.00 | 0.00 | 0.00 | 0.00 | 0.00 | 0.00 |
| Antiviral therapy | 0.00 | 20.00 | 0.00 | 0.00 | 0.00 | 0.00 | 0.00 |
| APTT | 0.00 | 50.00 | 0.00 | 0.00 | 0.00 | 0.00 | 0.00 |
| Arrhythmia | 0.00 | 100.00 | 0.00 | 0.00 | 0.00 | 0.00 | 0.00 |
| Aspartate aminotransferase | 50.00 | 70.59 | 50.00 | 66.67 | 0.00 | 0.00 | 0.00 |
| Autoimmune Diseases or Immunodeficiency | 0.00 | 50.00 | 0.00 | 0.00 | 0.00 | 0.00 | 0.00 |
| B Lymphocytes | 0.00 | 25.00 | 0.00 | 0.00 | 0.00 | 0.00 | 0.00 |
| Blood Coagulation Disorders | 0.00 | 0.00 | 50.00 | 0.00 | 0.00 | 0.00 | 0.00 |
| C-reactive protein | 100.00 | 94.12 | 66.67 | 0.00 | 0.00 | 0.00 | 100.00 |
| Cancer | 0.00 | 70.00 | 0.00 | 33.33 | 0.00 | 0.00 | 0.00 |
| Cardiac disease and injury | 0.00 | 0.00 | 33.33 | 0.00 | 0.00 | 0.00 | 0.00 |
| Cardiac troponin | 0.00 | 85.71 | 75.00 | 66.67 | 0.00 | 0.00 | 0.00 |
| Cardiovascular disease | 0.00 | 100.00 | 33.33 | 100.00 | 0.00 | 0.00 | 0.00 |
| CD4 | 0.00 | 20.00 | 0.00 | 0.00 | 0.00 | 0.00 | 0.00 |
| CD8 | 0.00 | 20.00 | 0.00 | 0.00 | 0.00 | 0.00 | 0.00 |
| Cerebrovascular disease | 0.00 | 71.43 | 0.00 | 0.00 | 0.00 | 0.00 | 0.00 |
| Chest tightness or Dyspnea | 0.00 | 92.00 | 0.00 | 100.00 | 0.00 | 50.00 | 50.00 |
| Chills | 0.00 | 66.67 | 0.00 | 0.00 | 0.00 | 0.00 | 0.00 |
| Chinese Medicine | 0.00 | 50.00 | 0.00 | 0.00 | 0.00 | 0.00 | 0.00 |
| Chronic kidney disease | 0.00 | 100.00 | 66.67 | 0.00 | 0.00 | 0.00 | 0.00 |
| Chronic liver disease | 0.00 | 50.00 | 0.00 | 0.00 | 0.00 | 0.00 | 0.00 |
| Chronic obstructive lung disease | 0.00 | 100.00 | 0.00 | 100.00 | 0.00 | 0.00 | 0.00 |
| CK-MB | 50.00 | 20.00 | 33.33 | 0.00 | 0.00 | 0.00 | 0.00 |
| Co-infection of Fungus | 0.00 | 100.00 | 0.00 | 0.00 | 0.00 | 0.00 | 0.00 |
| Co-infection of Other viruses | 0.00 | 50.00 | 0.00 | 0.00 | 0.00 | 0.00 | 0.00 |
| Comorbidity | 0.00 | 100.00 | 100.00 | 66.67 | 0.00 | 0.00 | 0.00 |
| Confusion or Fluster | 0.00 | 100.00 | 0.00 | 0.00 | 0.00 | 0.00 | 0.00 |
| Corticosteroid | 0.00 | 100.00 | 50.00 | 0.00 | 0.00 | 0.00 | 0.00 |
| Cough | 0.00 | 20.00 | 0.00 | 0.00 | 50.00 | 50.00 | 0.00 |
| Creatine kinase | 0.00 | 50.00 | 75.00 | 0.00 | 0.00 | 0.00 | 0.00 |
| Creatinine | 0.00 | 50.00 | 60.00 | 66.67 | 0.00 | 0.00 | 0.00 |
| Cumulative fluid balance in ICU | 0.00 | 0.00 | 100.00 | 0.00 | 0.00 | 0.00 | 0.00 |
| D-dimer | 100.00 | 86.67 | 100.00 | 100.00 | 0.00 | 0.00 | 0.00 |
| Diabetes | 100.00 | 73.33 | 66.67 | 33.33 | 0.00 | 0.00 | 0.00 |
| Diarrhea | 0.00 | 50.00 | 0.00 | 33.33 | 50.00 | 50.00 | 0.00 |
| Disseminated intravascular coagulation | 0.00 | 100.00 | 0.00 | 0.00 | 0.00 | 0.00 | 0.00 |
| Dry  Sore throat | 0.00 | 31.25 | 0.00 | 50.00 | 0.00 | 0.00 | 0.00 |
| ECMO | 0.00 | 100.00 | 100.00 | 0.00 | 0.00 | 0.00 | 0.00 |
| Electrolyte imbalance | 0.00 | 100.00 | 0.00 | 0.00 | 0.00 | 0.00 | 0.00 |
| Enlargement of lymph nodes or sinus | 0.00 | 100.00 | 0.00 | 0.00 | 0.00 | 0.00 | 0.00 |
| Erythrocyte sedimentation rate | 0.00 | 60.00 | 0.00 | 0.00 | 0.00 | 0.00 | 0.00 |
| Fever | 0.00 | 30.00 | 0.00 | 0.00 | 50.00 | 50.00 | 66.67 |
| Fibrinogen | 0.00 | 25.00 | 0.00 | 0.00 | 0.00 | 0.00 | 0.00 |
| Glucocorticoid therapy | 0.00 | 83.33 | 0.00 | 0.00 | 0.00 | 0.00 | 0.00 |
| Glucose | 0.00 | 50.00 | 0.00 | 0.00 | 0.00 | 0.00 | 0.00 |
| Headache or Dizziness | 0.00 | 36.84 | 0.00 | 25.00 | 50.00 | 0.00 | 0.00 |
| Hemoglobin | 0.00 | 0.00 | 0.00 | 50.00 | 0.00 | 0.00 | 0.00 |
| Hemoptysis | 0.00 | 100.00 | 0.00 | 0.00 | 0.00 | 0.00 | 0.00 |
| HR | 0.00 | 66.67 | 66.67 | 0.00 | 0.00 | 0.00 | 0.00 |
| Hypertension | 50.00 | 100.00 | 60.00 | 66.67 | 0.00 | 0.00 | 0.00 |
| IL-10 | 0.00 | 100.00 | 0.00 | 0.00 | 0.00 | 0.00 | 0.00 |
| IL-2 | 0.00 | 50.00 | 0.00 | 0.00 | 0.00 | 0.00 | 0.00 |
| IL-6 | 0.00 | 100.00 | 100.00 | 0.00 | 0.00 | 0.00 | 0.00 |
| International normalized ration | 0.00 | 50.00 | 0.00 | 0.00 | 0.00 | 0.00 | 0.00 |
| Intravenous immunoglobulin | 0.00 | 75.00 | 50.00 | 0.00 | 0.00 | 0.00 | 0.00 |
| Invasive mechanical ventilation | 0.00 | 100.00 | 80.00 | 100.00 | 0.00 | 0.00 | 0.00 |
| Lactate | 0.00 | 50.00 | 0.00 | 0.00 | 0.00 | 0.00 | 0.00 |
| Lactate dehydrogenase | 100.00 | 92.86 | 75.00 | 100.00 | 0.00 | 0.00 | 50.00 |
| Leucocytes | 0.00 | 75.00 | 0.00 | 0.00 | 0.00 | 0.00 | 0.00 |
| Liver disease | 0.00 | 66.67 | 50.00 | 0.00 | 0.00 | 0.00 | 0.00 |
| Mechanical ventilation | 0.00 | 100.00 | 50.00 | 0.00 | 0.00 | 0.00 | 0.00 |
| Myalgia or Fatigue | 50.00 | 39.29 | 0.00 | 0.00 | 0.00 | 0.00 | 0.00 |
| Myoglobin | 0.00 | 100.00 | 100.00 | 0.00 | 0.00 | 0.00 | 0.00 |
| Nausea or Vomiting | 0.00 | 46.67 | 33.33 | 50.00 | 0.00 | 0.00 | 0.00 |
| Neutrophil-to-Lymphocyte Ratio | 0.00 | 66.67 | 0.00 | 0.00 | 0.00 | 0.00 | 0.00 |
| Neutrophils | 100.00 | 64.71 | 50.00 | 100.00 | 0.00 | 0.00 | 50.00 |
| Non-invasive mechanical ventilation | 0.00 | 100.00 | 80.00 | 100.00 | 0.00 | 0.00 | 0.00 |
| NT-proBNP | 0.00 | 100.00 | 0.00 | 0.00 | 0.00 | 0.00 | 0.00 |
| Oseltamivir | 0.00 | 50.00 | 0.00 | 0.00 | 0.00 | 0.00 | 0.00 |
| Other disease | 0.00 | 100.00 | 100.00 | 0.00 | 0.00 | 0.00 | 0.00 |
| Oxygen Inhalation Therapy | 0.00 | 100.00 | 20.00 | 0.00 | 0.00 | 0.00 | 0.00 |
| Pneumonia | 0.00 | 50.00 | 0.00 | 0.00 | 0.00 | 0.00 | 0.00 |
| Pneumothorax | 0.00 | 100.00 | 0.00 | 0.00 | 0.00 | 0.00 | 0.00 |
| Procacitonin | 0.00 | 50.00 | 0.00 | 0.00 | 0.00 | 0.00 | 0.00 |
| Procalcitonin | 0.00 | 75.00 | 100.00 | 33.33 | 0.00 | 0.00 | 0.00 |
| Prothrombin time | 0.00 | 14.29 | 33.33 | 66.67 | 0.00 | 0.00 | 0.00 |
| Renal Insufficiency | 0.00 | 50.00 | 0.00 | 0.00 | 0.00 | 0.00 | 0.00 |
| Renal replacement therapy | 0.00 | 100.00 | 66.67 | 100.00 | 0.00 | 0.00 | 0.00 |
| Respiratory failure and disease | 0.00 | 80.00 | 50.00 | 0.00 | 0.00 | 0.00 | 0.00 |
| RR | 0.00 | 50.00 | 66.67 | 50.00 | 0.00 | 0.00 | 0.00 |
| Septic_shock | 0.00 | 100.00 | 0.00 | 0.00 | 0.00 | 0.00 | 0.00 |
| Serum amyloid A | 0.00 | 50.00 | 0.00 | 0.00 | 0.00 | 0.00 | 0.00 |
| Serum ferritin | 0.00 | 100.00 | 50.00 | 0.00 | 0.00 | 0.00 | 0.00 |
| Sex | 100.00 | 72.22 | 66.67 | 100.00 | 0.00 | 0.00 | 0.00 |
| Shock | 0.00 | 100.00 | 0.00 | 100.00 | 0.00 | 0.00 | 0.00 |
| Smoking | 0.00 | 33.33 | 50.00 | 0.00 | 0.00 | 0.00 | 0.00 |
| SOFA score | 0.00 | 0.00 | 100.00 | 0.00 | 0.00 | 0.00 | 0.00 |
| Sputum | 0.00 | 33.33 | 0.00 | 33.33 | 0.00 | 0.00 | 0.00 |
| Stuffy or Runny nose | 0.00 | 18.18 | 0.00 | 0.00 | 0.00 | 50.00 | 0.00 |
| Temperature | 0.00 | 22.22 | 0.00 | 0.00 | 0.00 | 0.00 | 0.00 |
| Thyroid Disease | 0.00 | 100.00 | 0.00 | 0.00 | 0.00 | 0.00 | 0.00 |
| TNF | 0.00 | 100.00 | 0.00 | 0.00 | 0.00 | 0.00 | 0.00 |
| Tonsil swelling | 0.00 | 100.00 | 0.00 | 0.00 | 0.00 | 0.00 | 0.00 |
| Total bilirubin | 50.00 | 36.36 | 66.67 | 100.00 | 0.00 | 0.00 | 0.00 |
| Urea nitrogen | 0.00 | 50.00 | 100.00 | 50.00 | 0.00 | 0.00 | 0.00 |
| White blood cell | 50.00 | 42.86 | 75.00 | 66.67 | 0.00 | 50.00 | 50.00 |

**Table S5. List of symptoms mentioned in social media**

| **Variable** | **Fraction of users (%)** | **Type** | **Synonyms*** |
| --- | --- | --- | --- |
| Abdominal pain | 0.17 | Rare | abdominal pain |
| Allergy like symptoms | 3.31 | Less Common | allergy like symptoms, seasonal allergies, allergic, Allergic, asthma allergies, bad asthma and allergies, Coronavirus allergies, coronavirus allergies, allergy |
| Anemia | 0.87 | Rare | anemia, anemic, Anemia, Anaemia, anaemia |
| Anorexia | 1.74 | Less Common | able to eat mostly full meals, appetite back, appetite is gone, healthy appetite, lack of appetite, loss of appetite, Lost my appetite, Poor appetite, regained my appetite, zero appetite, Loss of appetite |
| Anxiety | 17.77 | Common | agoraphobia, anxiety, anxiety attack, anxious, feeling anxious, high levels of anxiety, panic attacks, pessimistic anxious useless, social anxiet, social anxiety, stress anxiety, trouble finding outlets for my anxiousness, Anxiety, Anxious, Panic attacks, death anxiety COVID19, death anxiety covid19, bad anxiety |
| Arthritis | 1.22 | Less Common | arthritis, psoriatic arthritis, rheumatic condition, rheumatoid arthritis, arthralgia |
| Blood disorder | 1.92 | Less Common | bleeding, blood disorders, capillary bleeding, haemorrhaging, hemostatic, scurvy, hemoptysis |
| Body ache/pain | 26.66 | Common | ache, aches, acheyness, achy, achy feeling, active symptoms, back pain, backache, Bad aches, Bloody sore, body ache, body aches, body achess, body pain, Body still hurts, bodyaches, burning, burning pain, feeling of agony, Felt more aches, neck pain, neck pains, pain, painful, painful wisdom teeth, pains, quadriplegia, rib soreness, scratchy, sore, sprain, tight, twisted shit, whole body and eyes hurt, Pain, Body aches, Burning, cough bodyaches congestion chills, body aches headache stuffy, cough bodyaches, body aches stomach pain, chronic illness, Chronic pain, scratches, bad aches, bloody sore, aches sore, aches I |
| Chest congestion | 2.09 | Less Common | chest cold, chest congestion, congested, congestion, light congestion |
| Chest pain | 5.92 | Less Common | cardiac issues, chest back pain, chest pain, chest pain tightness, chest pains, chest pressure, heart condition, heart is distressed, heart palpitations, heart problems, shooting pains, Chest pains, Chest pain, CHEST PAIN, Chest Pains, cardiovascular events MI heart attack, bad chest and back pain |
| Chest tightness or Dyspnea | 19.69 | Common | able to breathe, able to breathe better, barely breath, barely out of breath, breath is labored, breathi, breathing a little rapidly, breathing deeply, breathing had improved, breathing is getting better, breathing is getting worse, breathing is much better, Breathing is not great, breathing is still bad, breathing is still not right, breathing issues, Breathing just sucks, breathing limited, breathing Ok, breathing problems, Breathing was getting scary and I was falling, breathing was more or less fine, Breathing your own breath, breathlessness, Breathlessness was frightening, bubbling sound when breathing, Can barely breath, "cant breath normally", cant breath, chest feels tight, chest tightness, could breathe fine, deep breathing, Difficulty breathing, dying breath, dyspnea, feeling of of the more unpleasant breath, hard time breathing, hard to breath, hard to breathe, heart flutter, heart is racing, heavy breathing, hot breath, hurting to breathe, hurts to breath, lack of normal breathing, literally stop breathing, lost my breath, palpitations, problems breathing, Short of breath, shortens of breath, shortness of breath, shortness of breath my symptoms, shortness of breath symptom, shortness of breath, stiff with my breathing, stop breathing, struggle to breathe, struggling for breath, struggling to breath properly, struggling to breathe, struggling to breathe wasn’t hard enough I managed to drop my bowl of hot ramen, tachycardia, tight chest, tightening, tightness, trouble breathing, unable to breath, short of breath, difficulty breathing, Shortness of breath, breathing ok, Chest tightness, breathing disease, breathlessness fatigue, fatigue shortness of breath, Shortness of breath Chest Pains, shortness of breath chest pains, chest tightness sore, bad breathing, breathing pain, breathlessly, confusion shortness of breath, shortness of breath Eye, shortness of breath eye pressure, Shortness of breath 2 20 Notes, shortness of breath 2 20 notes, Shortness of breath 4, Shortness of breath 4 20 Notes, shortness of breath 4 20 notes, Shortness of breath 4 20 Notes bad news I, Shortness of breath 6 20 Notes Feeling, Shortness of breath 8 20 Notes, shortness of breath 8 20 notes, shortness of breath but I, shortness of breath mostly, shortness of breath mostly I, shortness of breath tightness, shortness of breath., Shortness of breath. |
| Chills | 6.27 | Less Common | aches chills, bloody cold, body chills, Chill, chilling, chills, cold still, shivering, CHILLS, chill, Chills, chills aches, chills aches, cold chills |
| Cold like symptoms | 4.70 | Less Common | cold, Cold compresses, cold flu, cold going, cold like symptoms, cold symptoms, colds, Flu death, flu deaths, flu feeling, flu like symptoms, flu symptoms, seasonal flu, bad cold |
| Coma | 1.05 | Less Common | coma |
| Confusion or Fluster | 10.28 | Common | clear cognitive decline, cognitive decline, cognitive decline issues, cognitive dissonance, confused, confusion, Derangement Syndrome, disability DIS ABILITY to not make coherent thoughts, disoriented, dreamy eyes, "Dudes cognitive function", feel lost, hallucinations, hint of irony or self-awareness, lack of awareness, losing cognitive function, unconscious, zero self-awareness, Confusion, derangement syndrome |
| Constipation | 0.52 | Rare | constipated, Constipation, constipation |
| Cough | 34.49 | Common | barely coughing, cough, coughing, coughing attack, coughing spell, coughs, deep cough, dry cough, dry coughing, dry coughs, hacking cough, horrible coughing, Dry cough, Cough, Dry coughing, Coughing, DRY COUGH, covid cough, covid19 cough, covid19 and coughs all night and another who, Covid19 is tiredness coughing, covid19 is tiredness coughing, Cough chills fatigue, cough chills fatigue, cough chills headache runny nose, cough fatigue, cough headaches, headache cough, Headache cough, dry cough some aches, cough my chest hurt, dry cough chest tightness, cough difficulty breathing, coughs and shortness of breath, shortness of breath and cough, shortness of breath and dry cough, shortness of breath and dry coughs, coronavirus cough, Coronavirus cough, bad cough, cough I, cough runny nose, cough sore, Cough cough, cough cough, cough pneumonia, cough syrup, cough up tuesday, "cough", daytime cough, friday cough, Hi hacking cough, labored i cough, right barmy cough |
| Dehydration | 0.87 | Rare | Dehydrated, dehydration, dehydrated |
| Diarrhea | 3.14 | Less Common | diarrhea, diarrhoea, loose stools, runny poops, diarrhea sneeze sore, mouth diarrhea, toilet diarrhea |
| Dry or Sore throat | 12.89 | Common | light sore throat, sore throat, sore throat, strep throat, throat pain, tingling, Sore throat, runny nose sore throat, throat, Throat kinda tingling..., throats |
| Dry-related symptoms | 0.70 | Rare | dry, feels dry, hella thirsty, thirst |
| Dyssomnias | 5.40 | Less Common | able to sleep, awake, good sleep, hard to get to sleep, hard to sleep, insomnia, lack of sleep, Loosing sleep, loss of sleep, narcolepsy, Sleep annoyingly disrupted, sleep disorder, sleep paralysis, sleep weird, sleeping hydrating, sleeping disorder, sleeping tonight, Sleeps my biggest problem, sleepy lump, unable to sleep, s my sleep paralysis, sleepiness |
| Ear problem | 2.26 | Less Common | deaf, ear ache, ear infection, ear pain, earwax infection, misophonia, ringing in ears, inner ear infection, vertigo dizziness nausea ear pain, dumb deaf |
| Enlargement of lymph nodes or sinus | 0.70 | Rare | Hodgkins disease, swollen, swollen lymph nodes |
| Eye problem | 2.79 | Less Common | dirt clearly visible, dry eyes, eye pain, Eye pressure, eye strain, eye symptom, hurts to move her eyeballs, limited vision, poor vision, short sighted, tunnel vision, vision slightly blurry, visions, watery eyes, tired vision |
| Fever | 37.11 | Common | delirious, fever, fever brain, fever dream, fever dreams, fever sweats, fever symptom, feverish, fevers, hay fever, high fever, high fevers, high temperature, hot flashes, LASSA FEVER, low fever, Neck feels extremely hot, Night Fever, rising fever, up and down fever, Yellow Fever, Fever, Fevers, Lassa fever, Feverish, High fever, Yellow fever, fever the chills aches, fever aches chills, chills fever, fever chills, fevers chills, fever body aches cough fatigue, Fever cough fatigue aches, fever cough fatigue aches, Fever fatigue, fever fatigue, cough fever headaches, fever cough headache, fever headache, fever headache mouth ulcers, headache fever, fever shortness of breath body aches cough, fever body aches dry cough, fever body aches migraines sore, aches rising fever, fever aches, fevers aches, dry cough fevers shortness of breath and tightness, fever dry cough shortness of breath, fevers shortness of breath dry cough and weakness, breath fevers weakness, fever shortness of breath, shortness of breath and fever, Cough fever, cough fever, cough slight fever, coughing fever weakness, coughs and fevers, dry cough fever, Dry coughing Fever, fever cough, Fever cough, Coronavirus Symptoms Fever, coronavirus symptoms fever, baby fever, bad fever, cabin fever, fever 99.7ish, fever cold sweats, fever I, fever no sob, fever Prayers, fever sore, Fever 1793, fever alhamduli, fever atall, fever I, fever I start hallucinating, Fever shortness, fever shortness, fever skyrocket, fever w hallucinations, fever whisperer, Fever...., fevers symptoms, fevers I, fevers w hallucinations, lassa fever death, Slight fevers, Fever Dream, lassa fever |
| Gastrointestinal symptoms | 5.57 | Less Common | Belly is growling, bowel movement, bowels are back to normal, Crohns, feel something crawling out of my stomach, gas crisis, gastric problems, gastrointestinal symptoms, GI issues, GI problems, GI symptoms, hunger pain, hurt tummy, IBS, intestinal problems, loose bowels, selfishness is stomach turning, stomach aches, stomach cramps, stomach hurts, stomach issues, stomach pain, stomach ulcer, Thankfully bowels are behaving themselves, tight COVID19 crohnsdisease, tummy issues, Umbilical hernia, upset stomach, heart burn, Stomach ache, stomach ache, stomachache, chest stomach |
| Hair loss | 0.17 | Rare | lost all my hair, alopecia |
| Headache or Dizziness | 15.85 | Common | dizziness, dizzy, dizzyness, drowsiness, drowsy, head ache, head pain, headache, headache diseases, headaches, lightheadedness, migraine, migraines, pressure in my head, Headaches, Headache, Migraine, congestion headache fatigue, bad headache, bad headaches, runny nose headache, Slight headache, wheezing headache |
| Hemorrhoids | 0.35 | Rare | hemorrhoid, hemorrhoids |
| Hiccups | 0.52 | Rare | hiccups |
| Loss of smell or taste | 12.72 | Common | able to smell or taste, able to smell or taste anything, able to taste or smell, able to taste or smell anything, anosmia, cannot smell anything, cannot smell or taste, change in the way I tasted and smelled things, complete loss of sense of taste, complete loss of smell, complete loss of smell and taste, feeling no taste, impeccable taste, inability to taste in g, lack of ability to taste or smell, lack of smell and taste, lack of taste is often an early sign, limited taste, losing sense of taste and smell, Loss of sense of smell, loss of sense of smell and taste are definitely symptoms, loss of smell, loss of smell and taste, loss of taste, loss of taste and smell, lost all taste and appetite, lost my ability to taste, lost my sense of smell, lost my sense of smell and taste, lost my sense of taste, lost my taste and smell, lost our sense of smell and taste, Lost Sense of Smell, lost sense of smell and taste, lost sense of taste and smell bit nauseus, lost smell and taste, lost your smell or taste, metallic taste too, poor taste, sense of smell, sense of smell and taste, sense of smell and taste is so strange, sense of smell back, sense of smell has decreased, "sense of smell isnt", sense of smell or taste, sense of smell taste, sense of smell to return, sense of taste, sense of taste or smell, smell or taste, smell taste, taste smell, taste a little muted or off, taste and smell, taste and smell good, taste anything, taste buds happy, taste freshness, taste grape and copper and smell metal filings, taste in my mouth, taste n smell back, taste or smell, taste or smell anything, taste smell, taste so much better, taste the same, unable to taste food, Loss of smell and taste, loss of sense of smell, Loss of smell |
| Memory Disorders | 1.22 | Less Common | amnesia, memory loss, memory problems, short memory, Amnesia |
| Myalgia or Fatigue | 11.85 | Common | achilles injury, back cramps, Cramps, fatigue, fatigued, Fibromyalgia, muscle ache, muscle aches, muscle pain, muscle soreness, muscle twitching, muscle weakness, myalgia, numb, stiff, viral fatigue, cramps, Muscle aches, Fatigued, fibromyalgia, covid 19 social media fatigue, fatigue aches weakness, bad fatigue, fatigue weakness, muscle atrophy |
| Nausea or vomiting | 1.74 | Less Common | blood in my vomiting, vomit, vomited, vomiting, baby vomit |
| Nausea or Vomiting | 2.61 | Less Common | nausea, nauseated, nauseous, Nausea |
| No symptoms | 1.39 | Less Common | asymptomatic, asymptomatic disease |
| Oral problem | 0.87 | Rare | abrasions, dry mouth torture, mouth ulcers, Sensitive teeth, tooth ache |
| Respiratory symptoms | 2.26 | Less Common | clear lungs, deep lung pain, feel my lungs, lung is ours, lung issues well, Lung pain, lung parts being ripped loose, lung problems, lungs are clear, lungs are shallow, lungs hurt, lungs in good condition, respirators are easier, respiratory sympto, respiratory symptoms, lung pain |
| Skin problem | 4.36 | Less Common | acne, blemishes, blemishes shit, blisters, blood blister, bruised, chapped lips, dry skin, forehead acne, irritated, itch, itching, itchy, itchy feeling, Itchy feet, redness, sensitive ass bitch, sensitive skin, shingles, Spontaneous Urticaria, tickle, Itching, Urticaria, urticaria, rash |
| Sneezing | 2.79 | Less Common | fake sneezing, sneeze, sneezing, sneeze shaming, sneezes |
| Spasm | 0.35 | Rare | spasms |
| Sputum | 0.87 | Rare | phlegm in my throat, phlegm too, phlegm |
| Stuffy or Runny nose | 5.23 | Less Common | bloody nose, congested nose, mucus membranes, mucus secretions, nasal congestion, nose bleads, nose bleeds, runny nose, "Runny nose isnt coronavirus", runny noses, sinus drip, Sinus infecti, sinus infection, sinus symptoms, sinusitis, Slap her nose lightly, sniffles, stuffy nose, runny nose or sneezing, Runny nose |
| Suicidal Ideation | 1.74 | Less Common | Revolutionary Suicide, suicidal, suicidal depression, suicidal thoughts, suicide, suicide attempts, trying suicide, Suicide |
| Sweating | 2.61 | Less Common | cold sweats, hot cold sweats, night sweats, sweating, sweating profusely, sweats, Sweating, cold sweats nausea vomiting, wear sweats, blood sweat, palpitations sweating |
| Uncharacterized symptoms | 2.09 | Less Common | feels weird, malaise, strange feeling, strange symptom, Strangest symptom, weird feeling, weird symptom, weird symptoms, weirdest symptom, Feels weird |
| Urination problem | 0.35 | Rare | difficulty peeing, weakened bladder is ignored |
| Weakness | 27.35 | Common | cheeks weak, critically ill, crtically ill, debilitating symptoms, doing pretty well, doing well, equally well, exhausted, exhausted fast, faint, fallen ill, Falling well behind, falls well, fantastically well balanced, feel defeated, feel ill, feeling ill, Feeling like I was slowl, Feeling like shit, feeling much bette, Feeling much better, feeling much worse, feeling restless, feeling schadenfreude, feeling sick, Feeling sick and emo, feeling sick too, feeling the ill effects, feeling weak, feeling weird, feeling well, fell ill, felt generally unwell, Felt sick, heaviness, Ill, ill for well, ill I feel, ill sleep, illness, illnesses, infectious illness, lack of energy, lack of self awareness, lethargic, lethargy, limp reaction, long term illnesses, looked ill, looks physically ill, Losing all chill, physically and emotionally ill, physically disabled aswell, progressed well, recovering well, restlessness, ridiculously exhausted, "sad im sick", seriously ill, shaky energy, sick, sleepy, tired, tiredness, weak, weak I couldnt even walk, weak to move, weaker, weakness, Well I feel even more sick, zero energy, Tired, feeling much better, feeling like shit, TIRED, My weakness, restless, tiredness weakness |
| Weight gain | 0.52 | Rare | gained so much weight, weight gain |
| Weight loss | 1.22 | Less Common | lose so much weight so fast, lose some weight, losing weight still, lost 3 pounds, Lost 40lbs, lost 7 lbs, lost a lot about 30 pounds, lost weight |

* All synonyms that were mapped to a given variable were tabulated

**Table S6. Co-occurrence of symptoms in social media**

| **Symptom_A** | **Symptom_B** | **# of co-occurrence** |  | **Symptom_A** | **Symptom_B** | **# of co-occurrence** |
| --- | --- | --- | --- | --- | --- | --- |
| Fever | Cough | 639 |  | Anxiety | Ear problem | 3 |
| Fever | Body aches and pain | 305 |  | Chills | Respiratory symptoms | 3 |
| Fever | Chest tightness or Dyspnea | 301 |  | Cough | Hiccups | 3 |
| Headache or Dizziness | Cough | 299 |  | Anxiety | Arthritis | 3 |
| Weakness | Fever | 297 |  | Cough | Hair loss | 3 |
| Body aches and pain | Cough | 278 |  | Nausea or Vomiting | Respiratory symptoms | 3 |
| Cough | Chest tightness or Dyspnea | 258 |  | Allergy like symptoms | Confusion or Fluster | 3 |
| Weakness | Cough | 244 |  | Allergy like symptoms | Dyssomnias | 3 |
| Headache or Dizziness | Fever | 243 |  | Chest tightness or Dyspnea | Sputum | 3 |
| Headache or Dizziness | Body aches and pain | 157 |  | Chills | Skin problem | 3 |
| Weakness | Body aches and pain | 156 |  | Nausea or Vomiting | Ear problem | 3 |
| Body aches and pain | Chest tightness or Dyspnea | 136 |  | Confusion or Fluster | Respiratory symptoms | 3 |
| Weakness | Headache or Dizziness | 126 |  | Chest congestion | Confusion or Fluster | 3 |
| Cough | Anxiety | 117 |  | Chest tightness or Dyspnea | Enlargement of lymph nodes or sinus | 3 |
| Fever | Loss of smell and taste | 116 |  | Anxiety | Respiratory symptoms | 3 |
| Myalgia or Fatigue | Fever | 114 |  | Chest pain | Ear problem | 3 |
| Fever | Dry throat or Sore throat | 113 |  | Loss of smell and taste | Enlargement of lymph nodes or sinus | 3 |
| Cough | Loss of smell and taste | 107 |  | Body aches and pain | Spasm | 3 |
| Cough | Chest congestion | 106 |  | Anxiety | Sputum | 3 |
| Fever | Confusion or Fluster | 103 |  | Chest pain | Respiratory symptoms | 3 |
| Myalgia or Fatigue | Cough | 99 |  | Chest pain | Suicidal Ideation | 3 |
| Weakness | Chest tightness or Dyspnea | 98 |  | Skin problem | Ear problem | 3 |
| Fever | Anxiety | 93 |  | Body aches and pain | Arthritis | 3 |
| Chest tightness or Dyspnea | Anxiety | 90 |  | Loss of smell and taste | Nausea or Vomiting | 3 |
| Weakness | Anxiety | 90 |  | Loss of smell and taste | Blood disorder | 3 |
| Dry throat or Sore throat | Cough | 88 |  | Respiratory symptoms | Nausea or vomiting | 3 |
| Fever | Chills | 85 |  | Sweating | Eye problem | 3 |
| Body aches and pain | Loss of smell and taste | 85 |  | Respiratory symptoms | Anorexia | 3 |
| Stuffy or Runny nose | Cough | 80 |  | Chills | Anemia | 3 |
| Body aches and pain | Anxiety | 80 |  | Ear problem | Anorexia | 3 |
| Cough | Confusion or Fluster | 79 |  | Chest pain | Chills | 3 |
| Cough | Chest pain | 73 |  | Chest tightness or Dyspnea | Hair loss | 3 |
| Myalgia or Fatigue | Body aches and pain | 72 |  | Stuffy or Runny nose | Nausea or vomiting | 3 |
| Fever | Chest pain | 72 |  | Dehydration | Ear problem | 3 |
| Dry throat or Sore throat | Body aches and pain | 70 |  | Anxiety | Suicidal Ideation | 3 |
| Headache or Dizziness | Loss of smell and taste | 68 |  | Headache or Dizziness | Blood disorder | 3 |
| Headache or Dizziness | Chest tightness or Dyspnea | 67 |  | Diarrhea | Dyssomnias | 3 |
| Weakness | Dry throat or Sore throat | 66 |  | Headache or Dizziness | Sneezing | 3 |
| Body aches and pain | Chest pain | 65 |  | Dry throat or Sore throat | Dry-related symptoms | 3 |
| Weakness | Loss of smell and taste | 65 |  | Dry throat or Sore throat | Blood disorder | 3 |
| Myalgia or Fatigue | Chest tightness or Dyspnea | 63 |  | Weakness | No symptoms | 3 |
| Weakness | Myalgia or Fatigue | 62 |  | Weakness | Suicidal Ideation | 3 |
| Headache or Dizziness | Dry throat or Sore throat | 54 |  | Myalgia or Fatigue | Weird symptoms | 3 |
| Cough | Chills | 54 |  | Fever | No symptoms | 3 |
| Body aches and pain | Chills | 52 |  | Weakness | Coma | 3 |
| Fever | Stuffy or Runny nose | 52 |  | Myalgia or Fatigue | Blood disorder | 3 |
| Chest tightness or Dyspnea | Chest pain | 51 |  | Dry throat or Sore throat | Ear problem | 3 |
| Chest tightness or Dyspnea | Loss of smell and taste | 51 |  | Headache or Dizziness | Nausea or vomiting | 3 |
| Myalgia or Fatigue | Headache or Dizziness | 48 |  | Myalgia or Fatigue | Weight loss | 3 |
| Weakness | Chills | 46 |  | Myalgia or Fatigue | Anorexia | 3 |
| Headache or Dizziness | Chest congestion | 46 |  | Dry throat or Sore throat | Memory Disorders | 3 |
| Diarrhea | Cough | 45 |  | Gastrointestinal symptoms | Chills | 3 |
| Gastrointestinal symptoms | Cough | 42 |  | Dyssomnias | Sneezing | 2 |
| Headache or Dizziness | Anxiety | 41 |  | Chest tightness or Dyspnea | Coma | 2 |
| Dry throat or Sore throat | Chest tightness or Dyspnea | 41 |  | Dry throat or Sore throat | Skin problem | 2 |
| Fever | Chest congestion | 39 |  | Dry throat or Sore throat | Sputum | 2 |
| Stuffy or Runny nose | Body aches and pain | 39 |  | Confusion or Fluster | Weight gain | 2 |
| Weakness | Chest pain | 38 |  | Headache or Dizziness | Memory Disorders | 2 |
| Chest tightness or Dyspnea | Chills | 38 |  | Confusion or Fluster | Nausea or vomiting | 2 |
| Chest tightness or Dyspnea | Confusion or Fluster | 38 |  | Eye problem | Confusion or Fluster | 2 |
| Headache or Dizziness | Stuffy or Runny nose | 36 |  | Chest tightness or Dyspnea | Nausea or vomiting | 2 |
| Headache or Dizziness | Chest pain | 36 |  | Confusion or Fluster | Sneezing | 2 |
| Weakness | Confusion or Fluster | 32 |  | Dry throat or Sore throat | No symptoms | 2 |
| Cough | Nausea or Vomiting | 32 |  | Chest tightness or Dyspnea | Weight gain | 2 |
| Cough | Cold like symptoms | 32 |  | Dry throat or Sore throat | Chest congestion | 2 |
| Loss of smell and taste | Anxiety | 32 |  | Confusion or Fluster | Dry-related symptoms | 2 |
| Fever | Gastrointestinal symptoms | 32 |  | Weight loss | Anorexia | 2 |
| Myalgia or Fatigue | Loss of smell and taste | 30 |  | Chest tightness or Dyspnea | Weight loss | 2 |
| Headache or Dizziness | Gastrointestinal symptoms | 30 |  | Dry throat or Sore throat | Hiccups | 2 |
| Body aches and pain | Confusion or Fluster | 29 |  | Gastrointestinal symptoms | Eye problem | 2 |
| Fever | Cold like symptoms | 29 |  | Anxiety | Blood disorder | 2 |
| Dry throat or Sore throat | Loss of smell and taste | 28 |  | No symptoms | Suicidal Ideation | 2 |
| Weakness | Stuffy or Runny nose | 28 |  | Weird symptoms | Anorexia | 2 |
| Fever | Weird symptoms | 28 |  | Cough | Spasm | 2 |
| Headache or Dizziness | Chills | 27 |  | Chills | Sweating | 2 |
| Gastrointestinal symptoms | Body aches and pain | 27 |  | Chills | Chest congestion | 2 |
| Loss of smell and taste | Chills | 27 |  | Myalgia or Fatigue | Nausea or vomiting | 2 |
| Fever | Dyssomnias | 27 |  | Myalgia or Fatigue | Sneezing | 2 |
| Fever | Diarrhea | 25 |  | Chills | Blood disorder | 2 |
| Dry throat or Sore throat | Chest pain | 25 |  | Myalgia or Fatigue | Dehydration | 2 |
| Dry throat or Sore throat | Confusion or Fluster | 25 |  | Cough | Memory Disorders | 2 |
| Stuffy or Runny nose | Chest tightness or Dyspnea | 23 |  | Cough | Urination problem | 2 |
| Dry throat or Sore throat | Anxiety | 22 |  | Body aches and pain | Weight gain | 2 |
| Cough | Sweating | 22 |  | Chills | Nausea or vomiting | 2 |
| Fever | Respiratory symptoms | 22 |  | Myalgia or Fatigue | Memory Disorders | 2 |
| Weakness | Gastrointestinal symptoms | 22 |  | Anxiety | Anemia | 2 |
| Fever | Nausea or Vomiting | 22 |  | Chills | Enlargement of lymph nodes or sinus | 2 |
| Body aches and pain | Allergy like symptoms | 21 |  | Chest pain | Dehydration | 2 |
| Fever | Ear problem | 21 |  | Weakness | Hiccups | 2 |
| Body aches and pain | Cold like symptoms | 20 |  | Respiratory symptoms | Dehydration | 2 |
| Headache or Dizziness | Ear problem | 20 |  | Diarrhea | Spasm | 2 |
| Fever | Blood disorder | 20 |  | Loss of smell and taste | Sneezing | 2 |
| Fever | Allergy like symptoms | 19 |  | Eye problem | Dehydration | 2 |
| Fever | Eye problem | 19 |  | Loss of smell and taste | Memory Disorders | 2 |
| Myalgia or Fatigue | Dry throat or Sore throat | 19 |  | Allergy like symptoms | Sneezing | 2 |
| Fever | Skin problem | 19 |  | Loss of smell and taste | Dyssomnias | 2 |
| Diarrhea | Body aches and pain | 19 |  | Dry throat or Sore throat | Arthritis | 2 |
| Anxiety | Chills | 19 |  | Headache or Dizziness | Arthritis | 2 |
| Cough | Allergy like symptoms | 19 |  | Fever | Arthritis | 2 |
| Diarrhea | Chest tightness or Dyspnea | 19 |  | Fever | Dry-related symptoms | 2 |
| Gastrointestinal symptoms | Chest tightness or Dyspnea | 19 |  | Dry throat or Sore throat | Hair loss | 2 |
| Cough | Skin problem | 19 |  | Cold like symptoms | No symptoms | 2 |
| Cough | Sputum | 19 |  | Weakness | Memory Disorders | 2 |
| Cough | Dyssomnias | 19 |  | Chest pain | Confusion or Fluster | 2 |
| Headache or Dizziness | Respiratory symptoms | 19 |  | Diarrhea | Chills | 2 |
| Weakness | Cold like symptoms | 19 |  | Nausea or Vomiting | Confusion or Fluster | 2 |
| Body aches and pain | Chest congestion | 18 |  | Chest pain | Sputum | 2 |
| Dry throat or Sore throat | Stuffy or Runny nose | 18 |  | Skin problem | Arthritis | 2 |
| Myalgia or Fatigue | Confusion or Fluster | 18 |  | Diarrhea | Cold like symptoms | 2 |
| Fever | Dehydration | 18 |  | Cold like symptoms | Confusion or Fluster | 2 |
| Cough | Eye problem | 18 |  | Blood disorder | Dyssomnias | 2 |
| Myalgia or Fatigue | Anxiety | 17 |  | Blood disorder | Respiratory symptoms | 2 |
| Fever | Anorexia | 17 |  | Diarrhea | Anxiety | 2 |
| Chest pain | Anxiety | 17 |  | Gastrointestinal symptoms | Respiratory symptoms | 2 |
| Cough | Weight loss | 17 |  | Weakness | Anemia | 2 |
| Headache or Dizziness | Nausea or Vomiting | 17 |  | Cold like symptoms | Ear problem | 2 |
| Chest pain | Loss of smell and taste | 17 |  | Skin problem | Nausea or Vomiting | 2 |
| Cough | Anorexia | 16 |  | Chest tightness or Dyspnea | Oral problem | 2 |
| Body aches and pain | Ear problem | 16 |  | Eye problem | Memory Disorders | 2 |
| Headache or Dizziness | Diarrhea | 16 |  | Gastrointestinal symptoms | Blood disorder | 2 |
| Body aches and pain | Sweating | 15 |  | Stuffy or Runny nose | Respiratory symptoms | 2 |
| Anxiety | Confusion or Fluster | 15 |  | Nausea or Vomiting | Anorexia | 2 |
| Body aches and pain | Skin problem | 15 |  | Skin problem | Cold like symptoms | 2 |
| Myalgia or Fatigue | Gastrointestinal symptoms | 15 |  | Gastrointestinal symptoms | Cold like symptoms | 2 |
| Weakness | Diarrhea | 15 |  | Weakness | Urination problem | 2 |
| Weakness | Nausea or vomiting | 15 |  | Chest pain | Hiccups | 2 |
| Chest pain | Cold like symptoms | 14 |  | Stuffy or Runny nose | Skin problem | 2 |
| Headache or Dizziness | Confusion or Fluster | 14 |  | Weakness | Oral problem | 2 |
| Cough | Respiratory symptoms | 14 |  | Stuffy or Runny nose | Cold like symptoms | 2 |
| Stuffy or Runny nose | Chest pain | 14 |  | Sputum | Respiratory symptoms | 2 |
| Body aches and pain | Weird symptoms | 14 |  | Nausea or Vomiting | Nausea or vomiting | 2 |
| Body aches and pain | Anorexia | 14 |  | Myalgia or Fatigue | Constipation | 1 |
| Chest tightness or Dyspnea | Cold like symptoms | 14 |  | Sneezing | Memory Disorders | 1 |
| Fever | Coma | 14 |  | Chest pain | Anorexia | 1 |
| Myalgia or Fatigue | Chest pain | 13 |  | Allergy like symptoms | Eye problem | 1 |
| Weakness | Anorexia | 13 |  | Allergy like symptoms | Cold like symptoms | 1 |
| Weakness | Skin problem | 13 |  | Allergy like symptoms | Sweating | 1 |
| Loss of smell and taste | Chest congestion | 13 |  | Anxiety | Enlargement of lymph nodes or sinus | 1 |
| Body aches and pain | Eye problem | 13 |  | Myalgia or Fatigue | Coma | 1 |
| Body aches and pain | Dyssomnias | 13 |  | Allergy like symptoms | Respiratory symptoms | 1 |
| Body aches and pain | Respiratory symptoms | 13 |  | Allergy like symptoms | Dehydration | 1 |
| Loss of smell and taste | Weird symptoms | 13 |  | Chest pain | Dry-related symptoms | 1 |
| Headache or Dizziness | Cold like symptoms | 13 |  | Chest pain | Hair loss | 1 |
| Stuffy or Runny nose | Loss of smell and taste | 12 |  | Allergy like symptoms | Chest congestion | 1 |
| Fever | Sweating | 12 |  | Headache or Dizziness | Anemia | 1 |
| Headache or Dizziness | Skin problem | 12 |  | Dehydration | Weight loss | 1 |
| Cough | Dehydration | 12 |  | Fever | Memory Disorders | 1 |
| Cough | Nausea or vomiting | 12 |  | Loss of smell and taste | Nausea or vomiting | 1 |
| Chest tightness or Dyspnea | Nausea or Vomiting | 11 |  | Headache or Dizziness | Oral problem | 1 |
| Diarrhea | Gastrointestinal symptoms | 11 |  | Loss of smell and taste | Weight gain | 1 |
| Weakness | Ear problem | 11 |  | Weight loss | Nausea or vomiting | 1 |
| Headache or Dizziness | Sputum | 11 |  | Allergy like symptoms | Memory Disorders | 1 |
| Headache or Dizziness | Dehydration | 11 |  | Loss of smell and taste | Suicidal Ideation | 1 |
| Weakness | Respiratory symptoms | 11 |  | Loss of smell and taste | Arthritis | 1 |
| Loss of smell and taste | Allergy like symptoms | 11 |  | Loss of smell and taste | Dry-related symptoms | 1 |
| Chest tightness or Dyspnea | Sweating | 11 |  | Anxiety | Sweating | 1 |
| Myalgia or Fatigue | Stuffy or Runny nose | 11 |  | Headache or Dizziness | Dry-related symptoms | 1 |
| Chest tightness or Dyspnea | Chest congestion | 11 |  | Headache or Dizziness | Enlargement of lymph nodes or sinus | 1 |
| Fever | Weight loss | 11 |  | Headache or Dizziness | Urination problem | 1 |
| Body aches and pain | Dehydration | 10 |  | Headache or Dizziness | Weight loss | 1 |
| Chest tightness or Dyspnea | Allergy like symptoms | 10 |  | Headache or Dizziness | Weight gain | 1 |
| Headache or Dizziness | Anorexia | 10 |  | Headache or Dizziness | Hiccups | 1 |
| Cough | Blood disorder | 10 |  | Headache or Dizziness | Abdominal pain | 1 |
| Cough | Anemia | 10 |  | Myalgia or Fatigue | Oral problem | 1 |
| Anxiety | Skin problem | 10 |  | Dehydration | Nausea or vomiting | 1 |
| Weakness | Dyssomnias | 10 |  | Headache or Dizziness | Hair loss | 1 |
| Cough | Ear problem | 10 |  | Fever | Abdominal pain | 1 |
| Dry throat or Sore throat | Chills | 10 |  | Dehydration | Anorexia | 1 |
| Headache or Dizziness | Dyssomnias | 9 |  | Weakness | Sputum | 1 |
| Cough | Suicidal Ideation | 9 |  | Fever | Constipation | 1 |
| Weakness | Nausea or Vomiting | 9 |  | Myalgia or Fatigue | Hair loss | 1 |
| Fever | Nausea or vomiting | 9 |  | Sweating | Blood disorder | 1 |
| Chest tightness or Dyspnea | Respiratory symptoms | 9 |  | Weight gain | Anemia | 1 |
| Cough | Sneezing | 9 |  | Skin problem | Constipation | 1 |
| Weakness | Weight loss | 9 |  | Nausea or Vomiting | Weight loss | 1 |
| Body aches and pain | Nausea or vomiting | 9 |  | Skin problem | Dehydration | 1 |
| Anxiety | Cold like symptoms | 9 |  | Eye problem | Oral problem | 1 |
| Anxiety | Anorexia | 8 |  | Eye problem | Anemia | 1 |
| Chest tightness or Dyspnea | Skin problem | 8 |  | Eye problem | Coma | 1 |
| Gastrointestinal symptoms | Loss of smell and taste | 8 |  | Nausea or Vomiting | Enlargement of lymph nodes or sinus | 1 |
| Dry throat or Sore throat | Dyssomnias | 8 |  | Eye problem | Ear problem | 1 |
| Body aches and pain | Nausea or Vomiting | 8 |  | Eye problem | Sneezing | 1 |
| Chest tightness or Dyspnea | Suicidal Ideation | 8 |  | Confusion or Fluster | Hiccups | 1 |
| Headache or Dizziness | Allergy like symptoms | 8 |  | Confusion or Fluster | No symptoms | 1 |
| Anxiety | Allergy like symptoms | 8 |  | Eye problem | Dyssomnias | 1 |
| Loss of smell and taste | Anorexia | 8 |  | Eye problem | Nausea or Vomiting | 1 |
| Chest tightness or Dyspnea | Eye problem | 8 |  | Confusion or Fluster | Ear problem | 1 |
| Dry throat or Sore throat | Allergy like symptoms | 8 |  | Confusion or Fluster | Anorexia | 1 |
| Anxiety | Nausea or vomiting | 8 |  | Gastrointestinal symptoms | Skin problem | 1 |
| Chest tightness or Dyspnea | Blood disorder | 7 |  | Skin problem | Anorexia | 1 |
| Gastrointestinal symptoms | Anxiety | 7 |  | Nausea or Vomiting | Dehydration | 1 |
| Chest tightness or Dyspnea | Anemia | 7 |  | Confusion or Fluster | Memory Disorders | 1 |
| Chest pain | Allergy like symptoms | 7 |  | Nausea or Vomiting | Dyssomnias | 1 |
| Chills | Cold like symptoms | 7 |  | Cold like symptoms | Nausea or vomiting | 1 |
| Cough | Weird symptoms | 7 |  | Cold like symptoms | Anemia | 1 |
| Weakness | Allergy like symptoms | 7 |  | Cold like symptoms | Arthritis | 1 |
| Fever | Sneezing | 7 |  | Cold like symptoms | Dry-related symptoms | 1 |
| Cough | Dry-related symptoms | 7 |  | Cold like symptoms | Urination problem | 1 |
| Stuffy or Runny nose | Chest congestion | 7 |  | Cold like symptoms | Hiccups | 1 |
| Cough | Arthritis | 7 |  | Cold like symptoms | Respiratory symptoms | 1 |
| Chest congestion | Nausea or Vomiting | 7 |  | Sputum | Nausea or Vomiting | 1 |
| Weakness | Arthritis | 7 |  | Sputum | Confusion or Fluster | 1 |
| Weakness | Sweating | 7 |  | Blood disorder | Oral problem | 1 |
| Dry throat or Sore throat | Cold like symptoms | 7 |  | Blood disorder | Weight loss | 1 |
| Headache or Dizziness | Sweating | 7 |  | Blood disorder | Dehydration | 1 |
| Myalgia or Fatigue | Chills | 7 |  | Blood disorder | No symptoms | 1 |
| Anxiety | Chest congestion | 6 |  | Blood disorder | Nausea or Vomiting | 1 |
| Loss of smell and taste | Confusion or Fluster | 6 |  | Skin problem | Enlargement of lymph nodes or sinus | 1 |
| Loss of smell and taste | Cold like symptoms | 6 |  | Confusion or Fluster | Hemorrhoids | 1 |
| Loss of smell and taste | Skin problem | 6 |  | Confusion or Fluster | Anemia | 1 |
| Respiratory symptoms | Ear problem | 6 |  | Weird symptoms | Chills | 1 |
| Chest congestion | Sputum | 6 |  | Sweating | Chest congestion | 1 |
| Myalgia or Fatigue | Sweating | 6 |  | Respiratory symptoms | Anemia | 1 |
| Weakness | Eye problem | 6 |  | Chills | Anorexia | 1 |
| Gastrointestinal symptoms | Confusion or Fluster | 6 |  | Chills | Ear problem | 1 |
| Myalgia or Fatigue | Diarrhea | 6 |  | Chills | Weight loss | 1 |
| Myalgia or Fatigue | Skin problem | 6 |  | Chills | Dehydration | 1 |
| Weakness | Dehydration | 6 |  | Chills | Weight gain | 1 |
| Stuffy or Runny nose | Sneezing | 6 |  | Myalgia or Fatigue | Hiccups | 1 |
| Weird symptoms | Confusion or Fluster | 6 |  | Myalgia or Fatigue | Weight gain | 1 |
| Fever | Enlargement of lymph nodes or sinus | 6 |  | Myalgia or Fatigue | No symptoms | 1 |
| Weakness | Sneezing | 6 |  | Chills | Sputum | 1 |
| Fever | Anemia | 6 |  | Chills | Eye problem | 1 |
| Chest tightness or Dyspnea | Dehydration | 6 |  | Weird symptoms | Coma | 1 |
| Fever | Sputum | 6 |  | Weird symptoms | Dyssomnias | 1 |
| Weakness | Dry-related symptoms | 6 |  | Weird symptoms | Eye problem | 1 |
| Dry throat or Sore throat | Sneezing | 5 |  | Weird symptoms | Chest congestion | 1 |
| Stuffy or Runny nose | Anxiety | 5 |  | Respiratory symptoms | Suicidal Ideation | 1 |
| Stuffy or Runny nose | Nausea or Vomiting | 5 |  | Sweating | Confusion or Fluster | 1 |
| Stuffy or Runny nose | Confusion or Fluster | 5 |  | Confusion or Fluster | Arthritis | 1 |
| Anorexia | Nausea or vomiting | 5 |  | Sweating | Dyssomnias | 1 |
| Weakness | Blood disorder | 5 |  | Dyssomnias | Respiratory symptoms | 1 |
| Dry throat or Sore throat | Eye problem | 5 |  | Dyssomnias | Weight gain | 1 |
| Body aches and pain | Sneezing | 5 |  | Dyssomnias | No symptoms | 1 |
| Body aches and pain | Memory Disorders | 5 |  | Weakness | Constipation | 1 |
| Body aches and pain | Enlargement of lymph nodes or sinus | 5 |  | Myalgia or Fatigue | Abdominal pain | 1 |
| Gastrointestinal symptoms | Stuffy or Runny nose | 5 |  | Myalgia or Fatigue | Allergy like symptoms | 1 |
| Body aches and pain | Sputum | 5 |  | Chest congestion | Cold like symptoms | 1 |
| Gastrointestinal symptoms | Sweating | 5 |  | Dyssomnias | Nausea or vomiting | 1 |
| Diarrhea | Chest pain | 5 |  | Chest congestion | Skin problem | 1 |
| Chest tightness or Dyspnea | Ear problem | 5 |  | Chest congestion | Eye problem | 1 |
| Myalgia or Fatigue | Chest congestion | 5 |  | Dyssomnias | Memory Disorders | 1 |
| Anxiety | Nausea or Vomiting | 5 |  | Dyssomnias | Anemia | 1 |
| Anxiety | Eye problem | 5 |  | Sweating | Spasm | 1 |
| Diarrhea | Dry throat or Sore throat | 5 |  | Sweating | Nausea or vomiting | 1 |
| Diarrhea | Stuffy or Runny nose | 5 |  | Sweating | Ear problem | 1 |
| Loss of smell and taste | Weight loss | 5 |  | Fever | Urination problem | 1 |
| Chest pain | Skin problem | 5 |  | Myalgia or Fatigue | Enlargement of lymph nodes or sinus | 1 |
| Fever | Oral problem | 5 |  | Chest tightness or Dyspnea | Memory Disorders | 1 |
| Diarrhea | Sweating | 5 |  | Abdominal pain | Dry throat or Sore throat | 1 |
| Chills | Confusion or Fluster | 5 |  | Cough | Constipation | 1 |
| Loss of smell and taste | Eye problem | 5 |  | Abdominal pain | Stuffy or Runny nose | 1 |
| Skin problem | Respiratory symptoms | 5 |  | Chest tightness or Dyspnea | Dry-related symptoms | 1 |
| Fever | Suicidal Ideation | 5 |  | Dry throat or Sore throat | Anorexia | 1 |
| Myalgia or Fatigue | Respiratory symptoms | 5 |  | Cough | Weight gain | 1 |
| Allergy like symptoms | Chills | 5 |  | Suicidal Ideation | Anemia | 1 |
| Confusion or Fluster | Dyssomnias | 5 |  | Stuffy or Runny nose | Allergy like symptoms | 1 |
| Chest tightness or Dyspnea | Weird symptoms | 5 |  | Cough | Coma | 1 |
| Fever | Weight gain | 5 |  | Abdominal pain | Gastrointestinal symptoms | 1 |
| Myalgia or Fatigue | Ear problem | 5 |  | Nausea or vomiting | Oral problem | 1 |
| Headache or Dizziness | Eye problem | 5 |  | Chest tightness or Dyspnea | Spasm | 1 |
| Chest tightness or Dyspnea | Dyssomnias | 5 |  | Stuffy or Runny nose | Dry-related symptoms | 1 |
| Anxiety | Dry-related symptoms | 5 |  | Stuffy or Runny nose | Enlargement of lymph nodes or sinus | 1 |
| Loss of smell and taste | Sweating | 5 |  | Stuffy or Runny nose | Blood disorder | 1 |
| Chest tightness or Dyspnea | Hiccups | 5 |  | Stuffy or Runny nose | Eye problem | 1 |
| Anxiety | Weight loss | 5 |  | Arthritis | Dry-related symptoms | 1 |
| Loss of smell and taste | No symptoms | 4 |  | Weakness | Abdominal pain | 1 |
| Anxiety | Dyssomnias | 4 |  | Diarrhea | Ear problem | 1 |
| Sweating | Nausea or Vomiting | 4 |  | Body aches and pain | Blood disorder | 1 |
| Dry throat or Sore throat | Nausea or Vomiting | 4 |  | Diarrhea | Sneezing | 1 |
| Dry throat or Sore throat | Sweating | 4 |  | Anemia | Urination problem | 1 |
| Cough | Oral problem | 4 |  | Body aches and pain | Hiccups | 1 |
| Headache or Dizziness | Weird symptoms | 4 |  | Gastrointestinal symptoms | Arthritis | 1 |
| Myalgia or Fatigue | Eye problem | 4 |  | Cough | Enlargement of lymph nodes or sinus | 1 |
| Dry throat or Sore throat | Dehydration | 4 |  | Suicidal Ideation | Dry-related symptoms | 1 |
| Myalgia or Fatigue | Cold like symptoms | 4 |  | Stuffy or Runny nose | Spasm | 1 |
| Weakness | Enlargement of lymph nodes or sinus | 4 |  | Dry throat or Sore throat | Respiratory symptoms | 1 |
| Myalgia or Fatigue | Nausea or Vomiting | 4 |  | Chest tightness or Dyspnea | Urination problem | 1 |
| Myalgia or Fatigue | Dyssomnias | 4 |  | Body aches and pain | Hair loss | 1 |
| Weakness | Weird symptoms | 4 |  | Stuffy or Runny nose | Dehydration | 1 |
| Stuffy or Runny nose | Anorexia | 4 |  | Dry throat or Sore throat | Weight loss | 1 |
| Stuffy or Runny nose | Weight loss | 4 |  | Gastrointestinal symptoms | Chest pain | 1 |
| Anxiety | Oral problem | 4 |  | Fever | Hair loss | 1 |
| Gastrointestinal symptoms | Ear problem | 4 |  | Gastrointestinal symptoms | Chest congestion | 1 |
| Chills | Nausea or Vomiting | 4 |  | Constipation | Arthritis | 1 |
| Skin problem | Confusion or Fluster | 4 |  | Gastrointestinal symptoms | Dyssomnias | 1 |
| Chills | Dyssomnias | 4 |  | Gastrointestinal symptoms | Hiccups | 1 |
| Gastrointestinal symptoms | Nausea or Vomiting | 4 |  | Body aches and pain | Coma | 1 |
| Body aches and pain | Suicidal Ideation | 4 |  | Diarrhea | Abdominal pain | 1 |
| Cough | No symptoms | 4 |  | Body aches and pain | Hemorrhoids | 1 |
| Dyssomnias | Ear problem | 4 |  | Stuffy or Runny nose | Suicidal Ideation | 1 |
| Dry throat or Sore throat | Gastrointestinal symptoms | 4 |  | Gastrointestinal symptoms | Dehydration | 1 |
| Blood disorder | Confusion or Fluster | 4 |  | Chest pain | Nausea or Vomiting | 1 |
| Chest pain | Dyssomnias | 4 |  | Stuffy or Runny nose | Anemia | 1 |
| Body aches and pain | Dry-related symptoms | 4 |  | Abdominal pain | Cough | 1 |
| Body aches and pain | Oral problem | 4 |  | Diarrhea | Allergy like symptoms | 1 |
| Diarrhea | Loss of smell and taste | 4 |  | Gastrointestinal symptoms | Weird symptoms | 1 |
| Fever | Hiccups | 4 |  | Gastrointestinal symptoms | Anorexia | 1 |
| Body aches and pain | Weight loss | 4 |  | Dry throat or Sore throat | Coma | 1 |
| Loss of smell and taste | Dehydration | 3 |  | Chest pain | Chest congestion | 1 |
| Chest tightness or Dyspnea | Anorexia | 3 |  | Abdominal pain | Body aches and pain | 1 |
| Anxiety | Hiccups | 3 |  | Stuffy or Runny nose | Sweating | 1 |
| Anxiety | Weight gain | 3 |  | Weakness | Chest congestion | 1 |
| Stuffy or Runny nose | Chills | 3 |  | Diarrhea | Chest congestion | 1 |
| Chest tightness or Dyspnea | Sneezing | 3 |  | Diarrhea | Eye problem | 1 |
| Anxiety | Sneezing | 3 |  | Stuffy or Runny nose | Sputum | 1 |

**Table S7. Novel COVID-19 related symptoms**

| Symptoms | Literature | Social Media | Symptom type* |
| --- | --- | --- | --- |
| Weakness | 1 | 1 | Common |
| Chest tightness or Dyspnea | 1 | 1 | Common |
| Fever | 1 | 1 | Common |
| Confusion or Fluster | 1 | 1 | Common |
| Headache or Dizziness | 1 | 1 | Common |
| Cough | 1 | 1 | Common |
| Dry throat or Soar throat | 1 | 1 | Common |
| Myalgia or Fatigue | 1 | 1 | Common |
| No symptoms | 1 | 1 | Less Common |
| Chest congestion | 1 | 1 | Less Common |
| Chest pain | 1 | 1 | Less Common |
| Anorexia | 1 | 1 | Less Common |
| Coma | 1 | 1 | Less Common |
| Diarrhea | 1 | 1 | Less Common |
| Gastrointestinal symptoms | 1 | 1 | Less Common |
| Nausea or vomiting | 1 | 1 | Less Common |
| Chills | 1 | 1 | Less Common |
| Stuffy or Runny nose | 1 | 1 | Less Common |
| Nausea or Vomiting | 1 | 1 | Less Common |
| Blood disorder | 1 | 1 | Less Common |
| Arthritis | 1 | 1 | Less Common |
| Skin problem | 1 | 1 | Less Common |
| Enlargement of lymph nodes or sinus | 1 | 1 | Rare |
| Sputum | 1 | 1 | Rare |
| Anemia | 1 | 1 | Rare |
| Abdominal pain | 1 | 1 | Rare |
| Anxiety | 0 | 1 | Common |
| Loss of smell and taste | 0 | 1 | Common |
| Body aches and pain | 0 | 1 | Common |
| Allergy like symptoms | 0 | 1 | Less Common |
| Sweating | 0 | 1 | Less Common |
| Sneezing | 0 | 1 | Less Common |
| Dyssomnias | 0 | 1 | Less Common |
| Weight loss | 0 | 1 | Less Common |
| Memory Disorders | 0 | 1 | Less Common |
| Eye problem | 0 | 1 | Less Common |
| Respiratory symptoms | 0 | 1 | Less Common |
| Ear problem | 0 | 1 | Less Common |
| Weird symptoms | 0 | 1 | Less Common |
| Cold like symptoms | 0 | 1 | Less Common |
| Suicidal Ideation | 0 | 1 | Less Common |
| Urination problem | 0 | 1 | Rare |
| Weight gain | 0 | 1 | Rare |
| Dry-related symptoms | 0 | 1 | Rare |
| Hiccups | 0 | 1 | Rare |
| Hair loss | 0 | 1 | Rare |
| Hemorrhoids | 0 | 1 | Rare |
| Oral problem | 0 | 1 | Rare |
| Dehydration | 0 | 1 | Rare |
| Spasm | 0 | 1 | Rare |
| Constipation | 0 | 1 | Rare |
| Food allergy | 1 | 0 | - |
| Eructation | 1 | 0 | - |
| Conjunctival congestion | 1 | 0 | - |
| Tonsil swelling | 1 | 0 | - |
| Atopic dermatitis | 1 | 0 | - |
| Allergic rhinitis | 1 | 0 | - |
| Malaise | 1 | 0 | - |
| Drug hypersensitivity | 1 | 0 | - |

* Symptom types were determined based on the frequency of mention in social media data

Symptoms only observed in social media were defined as novel COVID-19 related symptoms.
